# Cancer neuroscience: signaling pathways and new therapeutic strategies for cancer

**DOI:** 10.1038/s41392-025-02364-y

**Published:** 2026-02-23

**Authors:** Sihui Zhang, Lin Yuan, Ping Lin, Gen Yang, Xikun Zhou, Jinfu Xu, Min Wu, Yongye Huang

**Affiliations:** 1 College of Life and Health Sciences, Northeastern University, Shenyang, China; 2 Wenzhou Institute, University of Chinese Academy of Sciences, Wenzhou, Zhejiang China; 3 Laboratory of Research in Parkinson’s Disease and Related Disorders, Health Sciences Institute, China Medical University, Shenyang, China; 4 Biological Science Research Center, Southwest University, Chongqing, China; 5 State Key Laboratory of Nuclear Physics and Technology, School of Physics, Peking University, Beijing, China; 6 Department of Biotherapy, Cancer Center & State Key Laboratory of Biotherapy, West China Hospital, Sichuan University, and Tianfu Jincheng Laboratory Chengdu, Jincheng, China; 7 Department of Respiratory and Critical Care Medicine, Shanghai Pulmonary Hospital, School of Medicine, Tongji University, Shanghai, China; 8 Department of Neurology, Medical College of Wisconsin, Milwaukee, WI USA; 9 Department of Medicine, Harvard Medical School and Brigham and Women’s Hospital, Boston, MA USA; 10 Key Laboratory of Bioresource Research and Development of Liaoning Province, College of Life and Health Sciences, Northeastern University, Shenyang, China

**Keywords:** Cancer microenvironment, Cancer microenvironment

## Abstract

From a neuroscience perspective, cancer neuroscience has emerged as a subfield of cancer research. Presumable mechanisms underlying cancer-related neuronal activity (termed neurosciences) include the induction and modulation of signaling pathways that govern cell fate determination and emotional responses (anxiety and stress), such as structural molecules (synaptic structures and current transduction) and secretory substances (neurotransmitters, cytokines, hormones and neuropeptides). In the past 3 years, these neuronal activities, which can either promote cancer growth or be hijacked by cancer cells to support tumor survival and invasion, have been widely demonstrated to be closely related to cancer progression. The molecular mechanisms are also being refined. Despite their great promise, translating neuroscientific discoveries into clinically actionable strategies for cancer diagnosis, prognosis, and treatment remains a formidable task. In this comprehensive review, we attempt to provide a full account of the intersection between neuroscience and cancer research. From the perspective of cancer neuroscience, we fully discuss the potential signaling molecules and their regulatory mechanisms, as well as targets and emerging therapeutic strategies that control tumor progression via multiomics approaches. Overall, cancer neuroscience may have unprecedented potential for understanding neuronal functions and cancer development, ultimately offering the significantly improved cancer treatment.

## Introduction

Neurons with approximately billion units are found throughout the body. The nervous system is the dominant regulatory system in the human body. In terms of function and location, the nervous system is categorically divided into two parts: the central nervous system (CNS) and the peripheral nervous system (PNS). Neural cell maturation, diversification, migration to specific anatomical locations, and functional circuit assembly, including axon pathfinding, synapse formation, and myelinated infrastructure establishment, are complex processes for nervous system circuit formation. Interestingly, cancerous brain cells exhibit certain features of the maturing nervous system, which is likely the initial driver of the early idea of cancer neuroscience.

In 2017, Venkatesh observed very rapid electrical activity in a population of human glioma cells.^1^ Cancerous brain cells can interact more actively with electrical communication between groups of brain cancer cells in a continuous and rapid manner.^2,3^ Similar approaches, such as the ability of tumors to recruit blood vessels to meet tumor tissues for their own feed and growth, cancer depends on the nervous system for almost everything to spread. Interestingly, neural involvement in previously neglected cancer research will exhibit unprecedented hidden regulatory effects. While deciphering which neurons and signaling pathways are involved, the immune system has emerged as a new participant in the interplay between oncology and neuroscience.^4^ Although this complexity poses challenges to unraveling biological mechanisms, the involvement of additional players may simultaneously open novel therapeutic avenues for patients.^5–8^

**Fig. 1:**
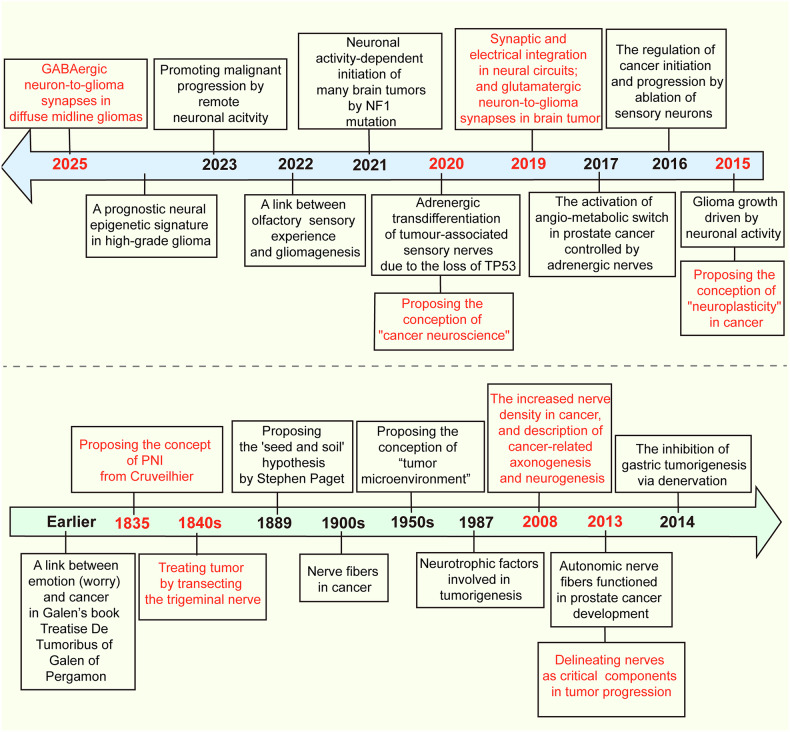
Representative discoveries of the cancer neuroscience aligned with timeline of historic milestones. A summary of the currently available milestones in neuro-cancer crosstalk, including but not limited to: proposing a link between emotion and cancer, confirming that nerves are components of tumor tissue, proposing neural plasticity, confirming that nerves regulate cancer progression, revealing that there is electrical communication and synaptic structure between nerves and brain tumors, etc. PNI perineural invasion, NF1 neurofibromatosis type 1

**Fig. 2:**
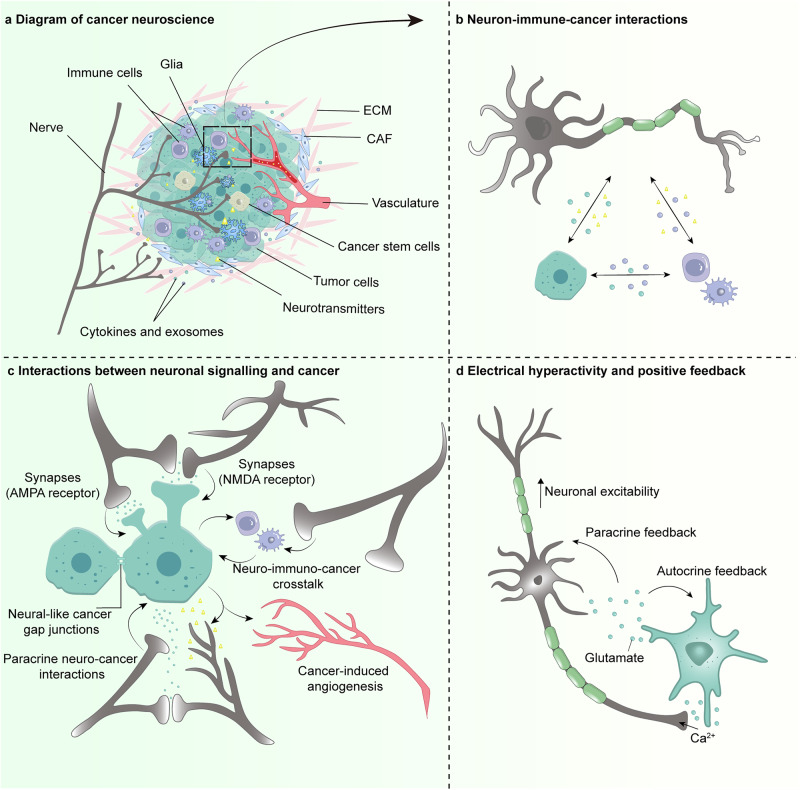
Schematic of cancer neuroscience—the core concept **a**. **b** Widespread and strong communication occurs in the crosstalk of neurons/nerves, immune cells and cancer cells. These interactions control immune cell trafficking and function, antitumor immunity, immune inhibition, and pro-cancer inflammation. **c** Neuronal activity regulates tumor malignant progression through paracrine growth factors secretion, neuro-glioma synapses, and Ca^2+^ influx currents. In turn, glutamate and synaptogenic proteins derived from glioma cells promotes neural hyperexcitability and circuits functional remodeling, thus generating excitatory neuronal activity which enhances proliferation and invasion to complete the autocrine feedback loop. **d** The overview of cancer neuroscience. There is nerve input into solid tumors (both in glioma and extracranial tumors), indicating the neuron/nerves participating in the interaction between various cell types, including cancer cells, stromal cells, and neurons in tumor microenvironment. Paracrine factors together with synapses signaling mediated by AMPAR and NMDAR regulate tumor proliferation and invasion, and in turn, tumor-derived factors promote cancer cells perineural invasion (PNI) and also peripheral nerves in growth into tumor microenvironment

Ideas and research related to cancer neuroscience were documented a long time ago, but their clear concept did not emerge until 2022 (Fig. 1). Treatise De Tumoribus of Galen of Pergamon was the earliest documented theory that tumorigenesis is associated with black bile, and that worry promotes the production of black bile, thus linking negative emotions to tumorigenesis. Some scholars regard it as the very beginning of cancer neuroscience. The report of nerve involvement was that breast cancer spread after migrating into and around the facial nerve by Cruveilhier in 1835, which represents the aggressive stage during breast cancer progression. Similarly, Alaya et al. reported that perineural invasion is an active interaction between nerves and prostate cancer cells.^9^ When the density of prostate cancer cells increases to the greatest number, more directional outgrowth of neurites from the dorsal root ganglia can be recruited into prostate cancer cell colonies and establish contact.^9^ Once contact is established, cancer cells travel along the nerves until they reach the neuronal cell bodies. Prostate cancer cell colony growth in dorsal root ganglia cell cocultures is greater than that in prostate cancer cell-only control cultures. Neurite outgrowth was also increased in coculture.^9,10^ The discovery of autonomic neurogenesis in prostate cancer tissue represents a breakthrough, and similar infiltration phenomena are emerging in other cancers, which all gradually accelerate the system development of cancer innervation mechanisms.^11,12^ On the basis of currently available results, interactions can be divided into two categories: indirect interactions, which are usually mediated by the recruitment of immune inflammatory cells, and direct contact through neural-tumor links, such as synapses. Neural signals activate the ion channels on tumor cells, inducing inward currents and establishing an electrical network among tumor cells. Additionally, tumor cells secrete glutamate, which not only creates an autocrine loop but also enhances neuronal excitability, and the secretion of neurotrophic factors further encourages axonal growth in neurons (Fig. 2).^6,13–16^

**Fig. 3:**
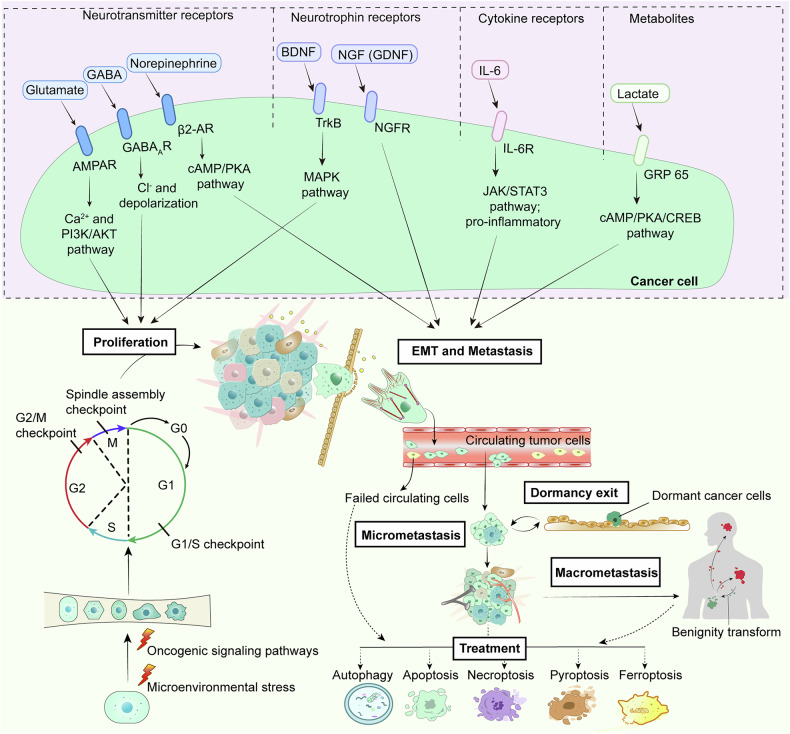
Mechanisms underlying cancer cell fate determination. Neural signaling molecular pathways determine the fate of cancer cells. Normal cells constantly undergoing proto-oncogene and tumor suppressor gene signaling pathway alternation and environmental stress (physical or chemical) transform into cancer cells. Subsequently, the loss of contact inhibition and proliferation restriction of cancer cells allows them indefinite proliferation. Growing up as the tumor, tumor cells continue to secrete signaling inducing angiogenesis to supply for nutrients and oxygen required of tumor tissues. Meanwhile, the cancer cells can be heterogeneous mediated by CSCs, and recruit normal stromal cells to evade immune attack. Subsequently, cancer cells will compress nearby tissues, generate MMPs, break through the basement membrane, produce morphological changes that undergo EMT, achieve extravasation into blood vessels or lymphatic vessels, and metastasis with a bias. After CTCs survive in the blood circulation reach distant metastatic sites, they extravasate to the appropriate niche. Some choose to form secondary tumors, while others may become dormant, evade surveillance, or even reactivate after treatment, causing cancer recurrence. Further micrometastasis grows to form macrometastasis, but certain tumor cells have been shown to be regulated to differentiate into benign cell lineages. Throughout the course of carcinogenesis and development, cancer cells under stress conditions, fail to survive in the blood circulation, and are attacked by the immune system or eliminated by treatment

The nervous system has a vital impact on cancer, functioning as a cooperator, in both brain tumors and peripheral tumors, with dominant outcomes of cancer progression.^15,17^ Not only do nerves provide a highway for the spread of cancer, but they also seem to create a safe protected area. The regulation of cancer through the nervous system can permeate the entire malignant process of cancer (Fig. 3 and Table 1). Tumor recurrence is a multifaceted process involving intricate cellular interactions within the tumor microenvironment and currently remains a significant challenge in brain tumor treatment. In isocitrate dehydrogenase (IDH)-wild-type and IDH-mutant glioma patients, tumors recur in distinct manners, which rely on the IDH mutation status and are attributed to changes in the histological feature composition, somatic alterations, and microenvironment interactions.^18^ IDH-wild-type tumors are more invasive at recurrence, and neoplastic cells exhibit elevated neuronal activities and characteristics, reflecting a possible role for neuronal interactions in promoting glioma progression.^18–20^ We emphasize multiple signaling pathways connecting cancer to neuroscience. In this review, we hope to contribute to research on nervous system innervation in tumor tissue progression, which may shed new light on the design of therapeutic strategies for cancer patients.

**Table 1:** Types of cell death modes in cancer and neurons

Cell death mechanisms	Mediators	Representative function in cancer	Representative function in neuroscience
Apoptosis (extrinsic)^373^	Caspases 8,3,6,7	Increase chemotherapy sensitivity;^374^ promote tumor cell survival^375^	Lead to ALS,^376^ AD,^377^ HD,^378^ PD^379^
Apoptosis (intrinsic)^373^	Caspases 9,3,6,7		
Necroptosis^380^	RIPK1,3; MLKL; TRADD; FADD	Inhibit tumor growth;^381^ promote tumor malignant progression^382^	Lead to neurodegenerative diseases;^383^ result in neuron and axonal degeneration^384^
Parthanatos^385^	PARP-1, PAR, AIF	Using PARP inhibitors to inhibit tumor progression^386,387^	Increased AIF activation and nuclear translocation in nervous system disorders;^388,389^ lead to neurotoxicity^390^
Ferroptosis^391^	Fe2+, ROS accumulation; lipid peroxidation	Enhance radiotherapy impact;^392^ inhibit tumor growth;^393^ influence surrounding healthy tissues^394^	Lead to neurodegeneration^395^
Pyroptosis^396^	Caspase-1,4,5,11; GSDMD	Double-edged sword^397^	Participate in the pathogenesis of neurodegenerative diseases, and exacerbate neuroinflammation and neurodegeneration^398,399^
Lysosomal cell death (LCD)^400^	Lysosomal membrane permeabilization, cathepsin; iron	Inhibit tumor growth and immune escape^401^	N/a
Autophagy^402^	ATG complex	Double-edged sword	Defects in autophagy participate in the pathogenesis of neurodegenerative diseases^395^
Disulfidptosis^403^	SLC7A11; NADPH;	Induce cancer cells death^403^	N/a
Cuproptosis^404^	ES and DSF; SLC31A1; DLAT; FDX1; Fe-S cluster	Participate in the mechanisms of tumorigenesis, metastasis, tumor immune escape^405^	Result in DA neuronal death in PD, and participate in PD pathogenesis^406^
Entosis^400^	CDH1; RHOA; ROCK; CDC42	Associate with the unfavorable prognosis and oncogenic characteristics^407^	N/a
NETosis^408^	NETs	Inhibit T-cell infiltration^409^	Harm the blood-brain barrier and neural cells, and enhance neuroinflammation^410^

## Crosstalk between the nervous system and cancer

**Fig. 4:**
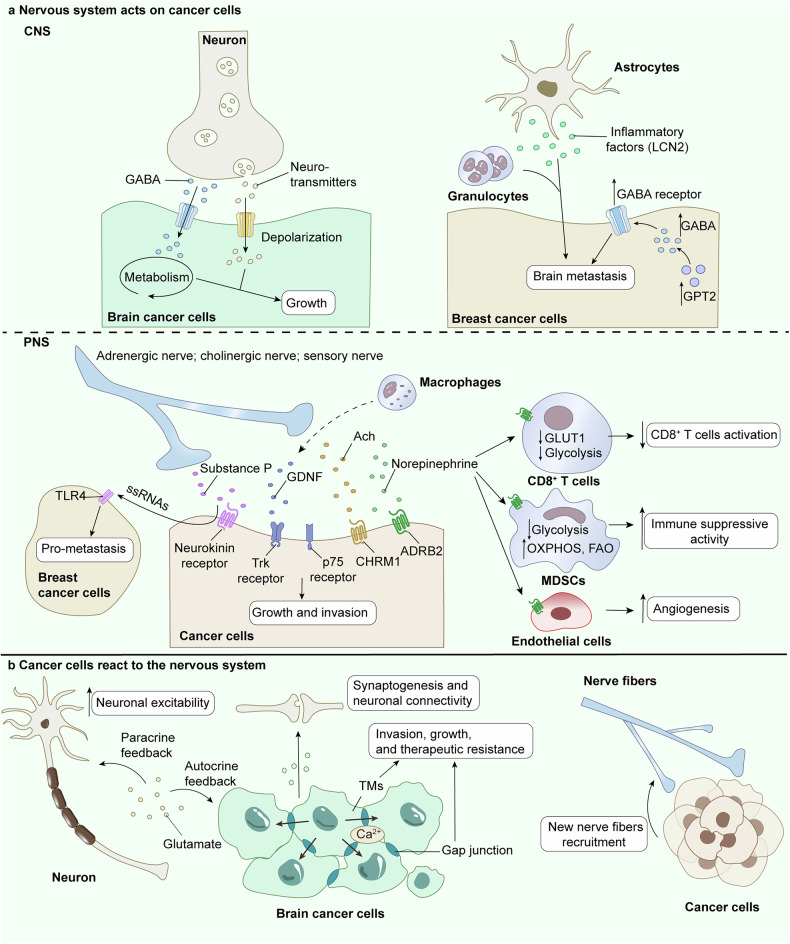
Interactions between nervous system and cancer. **a** Regulation of cancer cells by the CNS and PNS, respectively. In CNS, neurotransmitters from neuro-cancer synapses can induce depolarization of cancer cell membrane currents. Paracrine molecules can control tumor growth by manipulating cancer cell metabolism pathways. Astrocytes can release LCN2 to participate in chronic neuroinflammation by recruiting granulocytes, which promotes cancer brain metastasis. Further, the expression level of neurotransmitter receptors also modulates brain metastasis. In PNS, peripheral nerves (adrenergic nerve, cholinergic nerve, and sensory nerve) release neurotransmitters to modulate cancer malignant progression. **b** In turn, cancer can also act back on the nervous system, and this phenomenon occurs in both the CNS and PNS. In CNS, main manifestations include nervous system hyperactivity, synaptogenesis and neuronal connectivity. Glutamate secreted by tumor cells not only induces nerve excitability but also forms an autocrine loop to promote tumor tissue growth. In PNS, cancer cells can recruit new nerve fibers and promote innervation in the local TME

**Fig. 5:**
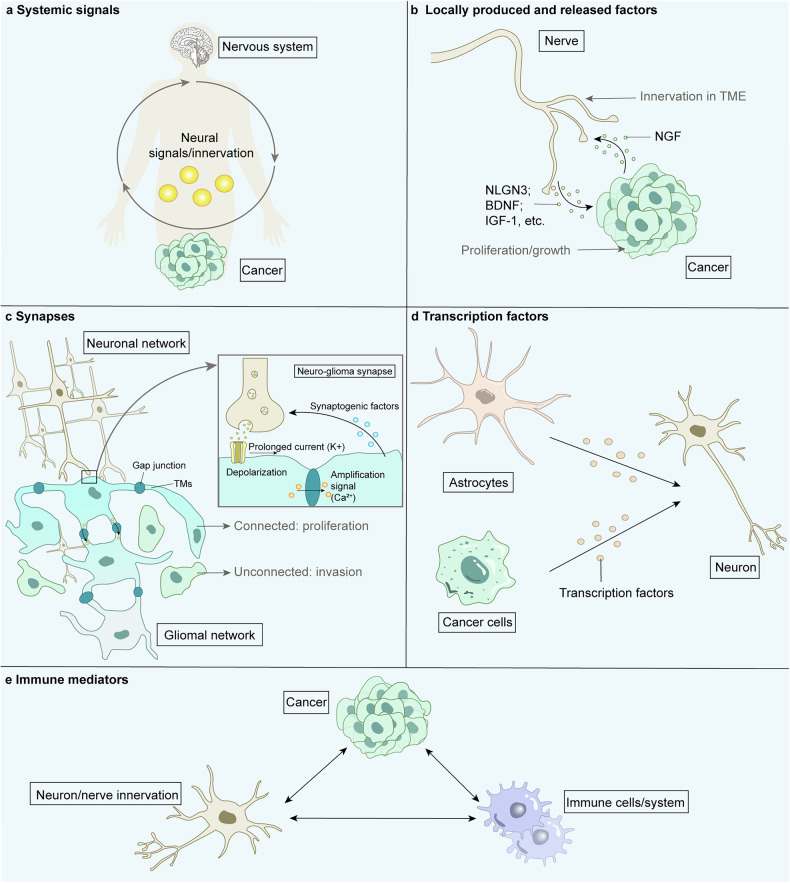
Molecules involved in nervous system-cancer interactions. The major molecules involved in the relationship between neural signaling and cancer cells‘ malignant progression include systemic signals (neurotransmitters and innervation) **a**, local signaling molecules (paracrine signaling) **b**, synapses (electrochemical signaling) **c**, transcription factors **d**, and immune mediators **e**

The communication between the nervous system and cancer is a fascinating topic. In this section, we classify and discuss the neuro-cancer crosstalk that has been demonstrated thus far (Figs. 4 and 5). Moreover, on the basis of the roles and preferences of classified signaling molecules, we speculate on signaling pathways associated with the key molecules involved in neuro-cancer communication.

### Secretory signals

Paracrine signal is one type of neuronal input. Neurons or nerve cells in the tumor microenvironment secrete neurotransmitters, neurotrophic factors, cytokines, and neuropeptides, and activate cancer-promoting pathways, such as the Wnt/β-catenin and STAT3 pathways, in tumor cells through receptors. Paracrine interactions in the tumor microenvironment have been described between neurons/nerves and cancer cells (Figs. 4 and 5).

#### Neuroligins (NLGN)

NLGN, a postsynaptic adhesion molecule that participates in synapse assembly and function, is cleaved and released into the tumor microenvironment to drive glioma cell proliferation.^15^ Secretion of NLGN3 by active neurons induces the PI3K-mTOR signaling pathway to promote glioma growth.^21^ An uncontrolled cell cycle is a condition in which cancer cells grow at an exceedingly fast pace. Certain cell cycle proteins are repurposed to serve functions outside of cell cycle control.^22^ CYY-1, a cyclin box-containing protein, drives synapse removal in this process. Moreover, CDK-5 facilitates new synapse formation by regulating the transport of synaptic vesicles to the sites of synaptogenesis. NLGN3, a Cdk5 substrate, is activated by Cdk5-mediated phosphorylation, which further augments NLGN3-mediated synaptic currents and synaptic transmission between neurons.^23^ CDK5 amplification may trigger the activation of STAT3, which is involved in tumor proliferation,^24^ suggesting that CDK5 may be at the critical crossroad between neuronal activity and tumor proliferation.

In addition to NLGN3, the neuronal activity-dependent signaling of BDNF and GRP78 promotes glioma proliferation and growth, acting as a neuronal activity-regulated glioma mitogen.^21,25,26^ NTRK2, a receptor of BDNF, is relevant to glioma proliferation and can be inhibited to decrease neuronal activity-induced proliferation.^26^ This function has also been demonstrated in neuroblastoma, as the overexpression of BDNF and GRP78 promotes proliferation, whereas their knockdown inhibits growth through the PI3K/AKT pathway.^27^ GRP78 can be regulated through the GRP78-FOXM1-KIF20A pathway to control proliferation and G2/M cell cycle arrest.^28^ Interestingly, neuronal re-entry into the cell cycle and cancer cell tumor suppression may activate the same p53-dependent signaling pathway to induce cell death. The cause of cancer cell proliferation likely involves alterations in the expression of cell cycle molecules. Abnormal activity of cyclin-CDK in stressed glial cells is also observed in glioma cells. This phenomenon suggests a considerable similarity between the mechanisms governing the normal division and differentiation of cells and those involved in carcinogenesis. An in-depth study of the processes of dendritic and axonal differentiation and maturation can provide clues for identifying neural communication targets in brain tumors.

NLGN3 transcription may also be regulated by the Wnt/β-catenin signaling pathway and surrounding secretions to transform neighboring cells into CSCs.^29^ These findings suggest that the function of Wnt/β-catenin is to regulate both NLGN3 expression and the acquisition of CSC properties in glioblastoma. The nuclear translocation of YBX1 has been shown to be closely linked to increased NLGN3 expression and glioblastoma progression.^30^ YBX1 binds to the NLGN3 promoter to increase its level through the nuclear import of NLGN3.^30^ In gastric cancer, YBX1 can regulate the transcriptional activity of the Wnt signaling pathway via long noncoding RNAs, which together form a positive feed-forward loop to promote gastric cancer malignancy.^31^ This phenomenon is not limited to the brain, as NLGN3 mutation and amplification have also been demonstrated to promote cancer growth-promoting functions via innervation.^32^ In peripheral cancers that exhibit YBX1-positive expression, NLGN3 expression can also be enhanced by YBX1 binding; however, it remains to be verified whether there is tissue specificity in the brain and peripheral cancers.

#### Nerve growth factor (NGF) and brain-derived neurotrophic factor (BDNF)

NGF and BDNF, which are neurotrophins secreted by pancreatic cancer cells, are tightly regulated by sympathetic innervation and enrichment of norepinephrine in the local microenvironment.^33^ These observations underscore the importance of cancer cell-derived neurotrophic growth factors as the main drivers of axonogenesis within the tumor and metastatic tumor program. Breast cancer has a brain metastasis bias through the ability of the abluminal vascular migration route to enter the CNS along the surface of emissary veins.^34^ Integrin α6, a laminin receptor expressed on the surface of breast cancer cells, helps them move along laminin-rich emissary veins to the leptomeninges. During this process, glial-derived neurotrophic factor (GDNF) plays an important role in reducing breast cancer cell death in nutrient-poor leptomeninges. GDNF is usually secreted by reactive CNS microglia and macrophages in response to brain injury, and it is located in the extracellular matrix (ECM) to block apoptotic neuronal stress responses. A majority of breast cancer cells colocalize with macrophages in meningeal leptomeninges, and stimulate macrophages to produce GDNF to promote the survival of brain metastatic cancer cells.^34^ Nerve secretion of GDNF and artemin promotes pancreatic cancer invasion.^35,36^ The PNS is highly relevant to the invasion and spread of primary peripheral tumors (Figs. 4 and 5). Perineural invasion (PNI), which has been observed for more than 100 years, is involved in cancer aggressiveness and poor survival rates.^12^ Sensory nerve innervation enhances breast cancer invasion and spread.^37^ β-adrenergic receptor-mediated sympathetic neural signaling can regulate cytoskeletal alterations and protease production in breast cancer to increase cancer invasion.^38^ In clinical samples, a new outcome emphasized that highly metastatic tumors in mouse models have higher densities of nerve fibers than do less metastatic tumors in tumor tissues, indicating that nerve densities are highly related to cancer progression and that cancer cells can induce the outgrowth of nerve fibers to increase the number of neural structures and nerve terminals in the local microenvironment.^39^ These findings suggest that tumor innervation and the vasculature are the major signals for tumors outside of brain metastases.

#### Insulin growth factor 1 (IGF1)

IGF1, a secreted paracrine signaling molecule stimulated by olfaction in an activity-dependent manner, mediates olfactory bulb gliomagenesis through sensory neuronal circuits,^40^ suggesting that specific sources of neuro-paracrine factors modulate the malignant characteristics of distinct cancer types. Several molecular markers of brain cancer have been shown to influence neurocognitive functions, such as psychomotor speed, memory performance, and executive function.^41^ These molecules influence cognitive performance via several possible mechanisms, such as the perturbation of neuronal communication. These findings may provide an entry point for a detailed discussion of the mechanisms by which cancer interferes with neural circuits to promote the malignancy.

#### Acetylcholine (ACh)

ACh, a classic neurotransmitter of axon terminals that is released by cholinergic nerves, can exert either activating or inhibiting signals and is also involved in tumor progression and spread. For example, denervation of cholinergic nerves or knockout of the receptor CHRM1 inhibits prostate tumor cell spread.^42^ Denervated stimulation of cholinergic nerves also leads to the progression of pancreatic cancer through the release of ACh. Ach activates the receptor CHRM1 in cancer stem cells, and CHRM1 signaling inhibits the downstream MAPK and PI3K/Akt pathways in pancreatic cancer cells.^42^ Sensory and sympathetic nerves promote the growth of pancreatic cancer cells through neurokinin receptors and adrenergic signaling, respectively, whereas parasympathetic nerves inhibit cancer stem cells through cholinergic signaling. These findings suggest that tumor innervation is not only very precise but also a balance of neuromodulation specificity. Additionally, ACh can suppress the expression of CCL5 in PDAC via histone deacetylation by histone deacetylase 1. Low levels of CCL-5 inhibit the immune effect of CD8^+^ T-cells, leading to an immunosuppressive TME.^43^ These findings suggest that endocrine signals can play a regulatory role in multiple fields of peripheral tumors.

#### Noradrenaline

Noradrenaline, known to trigger the angiogenic switch by ADRB2 (β-adrenergic receptor) in endothelial cells, can promote tumor growth.^44^ Adrenergic nerves indirectly regulate cancer cell survival through angiogenesis and metabolism for nutrient availability.^44^ Given that the initiation of angiogenesis is mediated by aerobic glycolysis in endothelial cells, sympathectomy and the conditional deletion of endothelial *Adrb2*, which encodes the β2 adrenergic receptor, can increase the expression of COA6, which is involved in the electron transport chain, and further mediate the metabolic shift from glycolysis toward oxidative phosphorylation to inhibit angiogenesis.^44^ Adrenergic nerve infiltration in the prostate cancer microenvironment leads to the release of norepinephrine from nerve terminals, which subsequently stimulates the expression of ADRβ2 in endothelial cells. The latter enables aerobic glycolysis in endothelial cells to control tumor angiogenesis and malignant progression. When *Adrb2* is ablated or inhibited, it not only activates neovascularization but also impairs tumor progression. The activation of β-adrenergic receptors located at the surface of cancer cells has been shown to promote tumor tissue growth by recruiting and nourishing nearby blood vessels.^45^ This broadens the field of angiogenesis research, as some phenotypic characteristics of endothelial cells or pericytes may be involved in the regulatory switch of angiogenesis in tumor tissues. Capillary pericytes in the vasculature play a key role in controlling the blood flow and diameter of capillaries throughout the brain.^46^ After vasoconstriction and slower blood flow are induced by optogenetic stimulation, capillary pericytes also show a slower rate of expansion close to baseline. When optogenetic stimulation is removed, capillaries dilate locally, and capillary blood flow increases in the absence of pericytes. Whether neural signals can also regulate pericytes is an urgent scientific question in vascular biology and tumor biology. The expression characteristics of pericytes, metabolic characteristics, and cytoskeletal contractility are related to the molecular mechanisms of neurotransmitter receptor expression or neuronal differentiation. There are specific alterations in the vasculature of healthy adults and in patients with brain tumors.^47^ How CNS-specific mechanisms control angiogenesis, whether this specificity can be compared and analyzed by multiomics in tumors, and how it is related to embryonic development and differentiation processes are still unanswered questions.

#### 5-Hydroxytryptamine (5-HT)

5-HT, also called serotonin, is released by enteric serotonergic neurons and combines with HTR1B/1D/1F receptors on the surface of colorectal cancer stem cells,^48^ which activates the Wnt/β-catenin signaling pathway to promote colorectal cancer stem cell self-renewal and tumorigenesis.^48^ In addition, the activity of serotonergic neurons can also regulate brain tumor formation by activating modifications to histones.^49^ After serotonylation on histones, the transcription factor ETV5 is enriched in ependymomas, which decreases overall survival and enhances tumor proliferation by downregulating the expression of neuropeptide Y.^49^ The overexpression of neuropeptide Y suppresses tumor progression by remodeling the brain microenvironment toward synaptic inhibition and, in turn, decreases brain hyperactivity and inhibits tumor progression.^49^ The activation of discrete subtypes of neurons suppresses ependymoma tumorigenesis, highlighting the need to decipher how neuronal-subtype and circuit-specific interactions affect brain-tumor progression.

#### Neuropeptides

Neuropeptides, important neural regulatory substances, are produced not only by the nervous system but also by the peripheral organs and are discussed in both the CNS and the PNS. Neuropeptides may function as neurotransmitters, neuromodulators, and endohormones. Paracrine signaling is also not limited to primary brain tumors.^34^ Breast cancer cells induce spontaneous calcium activity in sensory neurons and drive the release of the neuropeptide substance P from neurons. Elevated expression of substance P is associated with enhanced lymph node metastatic spread in patients. Substance P can act on cancer cell populations with high TACR1 expression, leading to the death of a small number of these cells. The dead cells then release single-stranded RNAs to act on TLR7 in neighboring tumor cells and thereby trigger the prometastatic gene expression program.^39^

### Synapses and electrical signals

#### Glutamate

Synaptic connections, which require synapse signals (e.g., glutamate), are critically pivotal for communication between neurons and their environments, where electrical activity may be used to control various cellular processes and behaviors, including newly generated neuron migration to the appropriate location and axonal targeting.^50–52^ The glioblastoma cell subpopulation with invasive features exhibited migration patterns similar to those of immature neurons. Bona fide synapse formation between presynaptic neurons and postsynaptic glioma cells drives tumor growth and invasion by transmitting electrical and chemical signals.^2,3,53^ These synapses are oriented from neurons to glioma cells via AMPA receptor-mediated excitatory postsynaptic currents in glioma cells. The use of AMPA receptor inhibitors can effectively reduce glioma cell proliferation and invasion.^3^ Another kind of synapse, similar to the position of tripartite synapses mediated by astrocytes, has been shown to be involved in breast-to-brain metastasis.^54^ The key element of these two types of synapses is the glutamate signal. Glutamate, a paracrine secreted factor, potentially plays a role in increasing neuronal hyperexcitability and tumor growth if abnormally produced in GABAergic interneurons in the glioblastoma microenvironment.^55,56^ BDNF paracrine secretion regulates neuron-to-glioma synapse number and AMPA receptor trafficking to the glioma cell membrane via the BDNF-TrkB pathway, thus increasing the amplitude of glutamate-evoked currents.^26^ Abrogation of BDNF secretion or blockade of glioma TrkB expression effectively decreases neuron-to-glioma synaptic connections, reduces neuronal activity-induced glioma proliferation, and prolongs survival.^26^ The secretion of NLGN3 also participates in regulating the number of neuron-to-glioma synapses.^2^ The health adaptability of synaptic plasticity, which emphasizes the interaction between neurons and gliomas, can be strengthened via an activity-dependent regulatory mechanism.

#### Gamma-aminobutyric acid (GABA)

According to the latest results, GABAergic inhibitory synapses exist in the first frontal diffuse midline gliomas, not only early recognized glutamatergic excitatory synapses.^57^ GABAergic input increases the intracellular chloride concentration and a strong inward current in diffuse midline glioma cells via the NKCC1 chloride transporter, leading to depolarization of the diffuse midline glioma cell membrane and promoting diffuse midline glioma cell proliferation. This once again demonstrates the important role of ion concentration differences and potential changes on the membrane surface of brain tumors in tumor development. However, in contrast to the above results, the loss of GABAergic neurons leads to the proliferation of tumor cells. The high chloride concentration in diffuse midline gliomas can also lead to depolarization of the cancer cell membrane, thereby promoting the survival of tumor cells. The strong inward current induced by GABAergic input is due to the difference in Cl^-^ concentration, which still leads to depolarization of the tumor cell membrane, thereby promoting brain tumor survival.^57^ We can speculate that membrane potential depolarization, rather than excitatory and inhibitory transmitters, is the main cause of cancer cell proliferation. The feature that tumor cells “hijack” neuronal features to support survival is worth exploring in peripheral tumors. An important breakthrough for combating and controlling malignant development and progression could be achieved if the brain metastasis of peripheral tumors mimics the hijacking of neural circuits and the generation and dissemination of electrical signals on the surface of the tumor cell membrane.

The overexpression of glutamic pyruvate transaminase (GPT2) elevates the concentration of GABA and then activates GABA_A_ receptors to promote breast cancer metastasis.^58^ In breast-to-brain metastatic models, the GABAergic phenotype is similar to that of neuronal cells. Breast-to-brain metastases take up and catabolize GABA into succinate, with the resulting formation of nicotinamide adenine dinucleotide as a biosynthetic source to provide proliferative ability itself.^59^ As a neurotransmitter, GABA is also a product of amino acid metabolism, and its expression and metabolic pathways may also be related to the trend of brain metastasis of tumors.

#### Neuronal excitability

Glioma cells have the ability to drive synaptogenesis^60,61^ and neuronal connectivity^62^ to further increase associated neuronal hyperexcitability. In brain tumor tissue, the tumor cells themselves exhibit neurodevelopmental features. Tumor microtubes (TMs) and tunneling nanotubes (TNTs), neurite-like membrane protrusions that mimic mechanisms of neurite pathfinding, are used to invade the brain and promote tumor colonization in glioma (Figs. 4 and 5).^63–65^ After neural stimulation, similar to immature neurons and oligodendrocyte precursor cells receiving message input from synapses, the invasion and the TMs genesis of glioma cells are increased.^53^ TMs act as routes for brain invasion and interconnection among tumor cells to form a resulting network in glioma.^66^ Single glioma cells are connected to functional and communication networks by TMs and gap junctions, which are essential for cancer metastasis, therapeutic resistance, and tumor homeostasis.^63,64,66^ Importantly, this type of connection is also found in the astrocytic network of the brain, which may provide resources for brain metastasis survival.^53^ Calcium transients and potassium currents that are spread by TMs and gap junction-mediated tumor networks have been shown to achieve electrical coupling, enable multicellular communication and drive brain tumor progression (Figs. 4 and 5).^2,3^ Indeed, accumulation of GAP43 and TTYH1 is observed at the tips of TMs, similar to the growth cones of neurites during neurodevelopment.^63,67^ In glioblastoma, a small number of tumor cells, called intrinsically rhythmic glioblastoma cells, exhibit autonomous activity, which generates rhythmic Ca^2+^ waves to influence tumor survival and growth.^68^ Along with the increased number of neuro-cancer synapses, the number of tumor-activating intercellular Ca^2+^ waves in glioma networks is also increased.^2,3^ These data suggest that electrical coupling allows electrical signals to propagate between cells and influence the behavior of tumor cells. In addition to considering synaptic structures, alterations in ion channels, neural plasticity, and cell connection coupling are also essential for broadening research directions. Some paracrine factors and synaptogenic factors are intermingled and result in glioma-induced neuronal hyperexcitability. In peripheral tumors, secreted neurotrophins released from nerves or secreted from tumor cells, have been shown to critically modulate tumor growth.^69,70^ The role of this peripheral nerve in the regulation of extrabrain tumors has been well described.^69,71^ Cancer outside of the brain can recruit new nerve fibers through axonogenesis to increase innervation and then remodel the neural microenvironment.^10,71^ The ability of peripheral tumors and recruited nerve fibers to develop electrical signal communication networks or metabolic networks similar to those of brain tumors may be a signal that triggers the survival and metastasis of tumor cell populations.

#### CGRP

Recent studies have demonstrated the existence of electrical signal transduction between peripheral nerves and peripheral tumors.^72^ Peripheral pain nerves (CGRP peptidergic neurons) are expanded in gastric cancer models in a manner dependent on tumor NGF expression, and there are anatomical and functional tight connections with tumor spheres. Through in vivo optogenetic and calcium imaging experiments, researchers have shown that activation of nociceptive nerves increases CGRP expression and release and the expression of the CGRP receptor RAMP1 on the tumor cell membrane. There is increased calcium flux between neurons and tumor cells.^72^ Bidirectional signaling between nerves and cancer cells promotes the proliferation of cancer cells and CAFs.

Nociceptive nerves also promote the metastasis of gastric cancer cells. Gastric cancer cells can migrate along the nerve, and then, circulating tumor cells can be detected in the portal vein.^72^ Transcription factors involved in neuronal reprogramming, such as NeuroD1 and ASCL1, are increased in cancer cells connected to neurons, suggesting that similar to glioma synapses, cancer cell-peptidergic neuronal circuits may induce neuron-like phenotypes in peripheral tumors promoting cancer metastasis. This finding highlights the importance of calcium communication in the peripheral tumor-nerve circuit and tumor cells with membrane potential depolarization and the upregulation of synaptophysin expression in cancer cells, suggesting that the communication mechanism between peripheral nerves and tumors is very similar to that in neuroglioma synapses. The mechanisms of sensory nerves and other types of peripheral tumors can also be further elucidated via similar approaches.

### Neurotransmitters and other chemical messengers or modulators

#### Lactic acid

Lactic acid (lactate), a product of glucose metabolism in tumor cells, plays an important role in the tumor microenvironment, not only serving as a metabolic substrate to support tumor cell survival and proliferation, but also acting as a signaling molecule with various biological effects. One of the key functions of lactic acid is its ability to stimulate angiogenesis to provide nutrients and oxygen for tumor cells, and enhance the production of enzymes that digest the ECM for cancer invasion.^73,74^ In addition to overcoming nutrient deficiency by activating metabolic mechanisms, lactate has been proven to remodel histone acetylation and regulate gene expression to promote GBM cell survival and proliferation.^75^ In the CNS, glial cells promote axon regeneration after CNS injury via the lactate-GABA_B_ receptor-cAMP signaling pathway.^76^ The lactate-GABA_B_ receptor-cAMP signaling pathway can increase the stability and growth of axons. Similarly, axon regeneration has also been revealed in the PNS, in which lactate metabolism in neurons enhances axon stability mediated by Schwann cells.^77^ In summary, lactate might regulate neural signals to control the development of GBM because of the beneficial effects of lactic acid on axon regeneration in the CNS and PNS. However, it remains unclear whether lactate promotes GBM cell survival through mediating axon generation in local tumor microenvironment of GBM. One study demonstrated that anabolic and proliferating cells perform biomass synthesis via incomplete oxidation of glucose and high glucose consumption.^78^ The photoreceptor cells located on the outer retina still produce lactate in the presence of oxygen, enabling diurnal renewal of the photoreceptor outer segments.^79^ Under hypoxic conditions, dysregulation of oxidative phosphorylation and the tricarboxylic acid cycle (TCA) cycle in rods with an activated hypoxic response decelerates cellular anabolism, causing shortening of rod photoreceptor outer segments before the onset of cell degeneration. An intact TCA cycle does not exhibit these early signs of anabolic dysregulation and shows a slower course of degeneration, which highlights the importance of mitochondrial metabolism, especially the TCA cycle, for photoreceptor survival in conditions of increased hypoxia-inducible factor activity.^80^ Lactate produced by astrocytes is exported to neurons to fuel mitochondrial respiration to support synaptic activity.^81^ Overactivation of the kynurenine signaling pathway by indoleamine 2,3-dioxygenase 1 (IDO1) causes astrocytes to fail to produce sufficient lactate as an energy source for neurons, thereby disrupting healthy brain metabolism and damaging synapses.^81^ An IDO1 inhibitor rescues the ability of astrocytes to produce lactate, which is subsequently taken up by neurons to provide energy for mitochondrial respiration and synaptic activity.^81^ These reports suggest that glucose metabolic pathways are critical for ensuring nervous system homeostasis and are important mechanisms for neuro-cancer communication.

#### Dopamine

Dopamine, a monoamine neurotransmitter, affects the interplay between cancer progression, immunity, and the central nervous system.^82^ The treatment of prolactinomas in pituitary neuroendocrine tumors is unique in that they are the only first-line treatment, which is achieved with dopamine agonist drugs. Through transcriptome sequencing and single-cell sequencing analysis of drug-resistant and drug-sensitive prolactinomas, DA resistance was shown to be associated with upregulation of the adhesion plaque (FA) signaling pathway. Fagin can exert its antitumor effect by inhibiting the expression of FA pathway components. Fagin inhibits the proliferation of pituitary tumor cell lines and organoids and promotes the apoptosis of pituitary tumor cells. In in vivo experiments, genistein effectively inhibited the formation of subcutaneous tumors.^83^ Dopamine is produced not only by the brain, but also by peripheral organs, such as the spleen and pancreas.^84,85^ Detections in lung cancer patients indicate significant elevation of plasma dopamine in malignancy due to stress from the disease process, which can inhibit T-cell proliferation and cytotoxic capacity.^86^ Interestingly, one study revealed an unconventional role for dopamine receptor (DRD4) beyond its classic function as a neurotransmitter receptor.^87^ DRD4 expression is significantly upregulated in colorectal cancer clinical samples, which is associated with poor patient prognosis. In vitro experiments have shown that simple overexpressed DRD4 can interact with transforming growth factor β receptors (TGFBR1 and TGFBR2) to induce epithelial-mesenchymal transition (EMT) in cancer cells independent of dopamine. The regulatory role of dopamine in malignant tumor progression has been described in detail in a recent reference.^85^ In this section, we discuss the latest dopamine-based technological innovations. The use of MgO_2_ nanoparticles and dopamine-conjugated gelatin as the main components for the clinical treatment of osteosarcoma, which can release H_2_O_2_ via photothermal therapy to significantly inhibit tumor recurrence, and sustainable Mg^2+^ effectively promotes bone regeneration, has been reported.^88^ Similarly, dopamine-carrying nanomaterials have also shown effective therapeutic capabilities, on the one hand depleting essential nutrients to cancer cells and curbing cancer cell survival and, on the other hand, near-infrared lasers allowing real-time fluorescence imaging and guiding dopamine-mediated mild photothermal therapy to resist tumors together.^89^

#### Melatonin

Melatonin, an amino acid hormone, is produced by the ingestion of tryptophan in the body, is converted to 5-hydroxytryptamine (serotonin) and is then converted to melatonin by N-acetyltransferase and hydroxyindole-O-methyltransferase in the pineal gland. Melatonin is a major neurotransmitter and neurohormone involved in transferring information to the CNS to regulate brain function, such as circadian rhythm and sleep, as well as exerting antitumor effects on various cancer types.^90,91^ The effects of melatonin on cell fate determination-related mechanisms may have therapeutic potential and may be further explored to address this open question.

### Transcription factors

Transcription factors are widely known to regulate gene expression. In our current understanding, they are more likely to reflect some similarity between neural component development and brain tumor progression (Fig. 5).

#### ZEB2

ZEB2 is a transcription factor that often functions in early germ layer specification and neocortical axon outgrowth, highlighting its importance in human neurodevelopment.^92,93^ In many aspects, the switch from neuroepithelial cells to transitioning neuroepithelial cells to radial glia resembles a partial EMT process with a progressive change in cell morphology, apical constriction, and a reduction in cell–cell junctions.^94^ Several reports have shown cross-regulation between ZEB2, the actin cytoskeleton and lipid metabolism.^95,96^ In glioma, ZEB has been confirmed to be involved in the regulation of its malignant progression, and there is an antagonistic effect with microRNAs to regulate the malignant invasion of glioma.^97^ Therefore, in other tumors with a propensity for brain metastasis, such as lung cancer, breast cancer, melanoma and colorectal carcinoma, investigating the molecular biological function of ZEB and epigenetic modulation molecules may reveal unknown effective metastasis regulatory pathways and targets for inhibiting cancer metastasis.

Studies have revealed the retinal cell development via single-cell RNA-seq and assays for transposase-accessible chromatin with high-throughput sequencing (ATAC-seq) analyses, indicating that the different subtypes of retinal neurons in vertebrates arise from distinct lineages.^98^ The discovery that a single transcription factor can efficiently reprogram neural precursor cells from a specific lineage into a particular type of retinal neuron represents a breakthrough in the field of cellular reprogramming.^98^

#### NeuroD1

NeuroD1 directly reprograms endogenous astrocytes into neurons, which can successfully develop and integrate into the visual cortical circuit after ischemic injury, leading to vision recovery.^99^ Reprogrammed astrocytes can even differentiate into spinal cord neurons and form synapses with host neurons upon transplantation, representing a major advance in reprogramming.^100^ NeuroD1 has also been shown to induce reprogramming from glioblastoma cells to glutamatergic neurons.^101^ Despite these advances, cross-lineage differentiation is controversial, and the differentiation in the nervous system is currently largely restricted to lineages.^102,103^ Reprogramming of cells into different lineages is likely difficult, and understanding the lineage-specific transcription factors and epigenetic modifications for reprogramming may be highly important. Astrocytes, for example, can reflect developmental cues because they are susceptible to reprogramming.^104^ Similarly, it is interesting to ask whether neurons that can be reprogrammed by astrocytes have conserved sequences in their genome and transcriptome sequences. It is not clear whether there are some program restrictions in lineages of benign intracellular transformation, and it is also unknown whether there are any characteristics of cancer cells that overcome this restriction.

### Neuro-immune-cancer circuit

The communication between the nervous and immune systems was first identified more than three decades ago and is now well established. The tumor microenvironment can be subdivided into several different categories and related drug repurposing, with the main concerns being hypoxia and lactic acid metabolism, lipid metabolism, pH, and innervation.^105^ In addition, in primary and recurrent glioblastoma resections of patients, the expression of hallmark genes of glioblastoma does not significantly change, but tumor purity decreases over time.^19^ Moreover, this change is accompanied by increased expression of neuron and oligodendrocyte marker genes in tumors.^19,106^ PNS, especially the autonomic nervous system, mainly participates in the transmission of instructions and inflammatory regulation of the tumor immune response outside the brain tumor.^107,108^ Sympathetic nerves, or adrenergic signals, regulate the migration and responsiveness of immune cells to control the strength of the immune susceptibility.^109^ In response to psychological stress, stress networks in brain regions induce peripheral lymphocyte and monocyte migration to a suitable functional landscape to calibrate the response of the immune system to physical threats.^110^ In addition, stress upregulates the expression of the glucocorticoid-inducible factor TSC22D3 and subsequently subverts anticancer immune therapy.^111^ Close relationships have been demonstrated among plasma cortisol levels, negative psychological mood, and tumor progression in cancer patients.^111,112^ It is also not surprising that sensory nerves can influence the immune response of solid tumors.

**Fig. 6:**
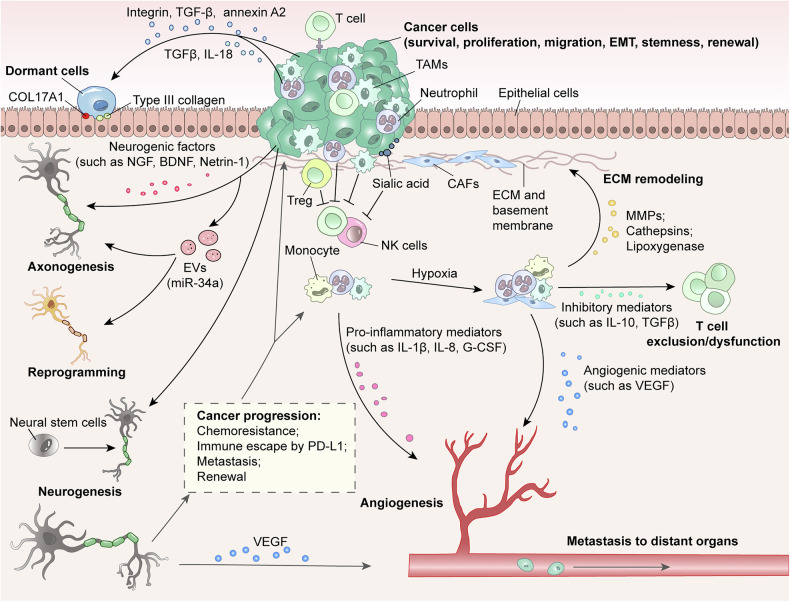
Crosstalk in neuron-immune-tumor microenvironment for cancer cell fate determination. The evolving tumor microenvironment is essential during all stages of cancer progression, supported by multiple key representative elements, including multiple immune cells (T-cell, Treg, NK cell, TAMs, neutrophil, and monocyte), CAFs, neurons, ECM, and cytokines. Normally, cancer cells are attacked to destruction by the immune system at the earliest stages of tumor initiation, while the immune system is gradually influenced along with the development of cancer to eventually provide pro-cancer functions. The hypoxic environment in which the cancer grows promotes immune cells and CAFs to secrete VEGF, IL-10, TGFβ, MMP-9, and integrin to support tumor growth, proliferation, angiogenesis, dormancy, EMT, invasion and metastasis. Meanwhile, neurons in tumor microenvironment are recruited to promote tumor malignant development, in turn, cancer cells secrete factors and EVs to regulate axongenesis, neuron reprogramming, and neurogenesis. (The arrows represent induct, and the horizontal lines represent inhibit)

The cancer cells in the tumor microenvironment might crosstalk with certain stromal cells, including CAFs, macrophages, MDSCs, and immune cells, as well as with vessels, nerves, and ECM.^113,114^ Cancer cells manipulate surrounding normal cells (cancer-associated fibroblasts, endothelial cells, and immune cells, etc.) or induce tumor microenvironment cells producing immunosuppressive cytokines, such as TGFβ, IL-10, or PGE2, to generate a growth-supportive microenvironment.^115^ Brain-gut axis is an essential pathway networks consisting of complex interdisciplinary fields including nervous system, immune system, and microbes, which actively participates in a variety of brain cancer types.^116^ Gut microbes, an important component of brain-gut axis, can secrete gut microbial neurotransmitter to mediate the malignant progress of tumor cells, which is similar the phenomenon in neuro-glioma in brain.^117,118^ The mechanisms, underlying cross-talk among immune cells, tumor cells and nervous system, profoundly influence tumor cells phenotype and behavior, as well as promote tumor proliferation, invasion, immune escape, and a more favorable microenvironment with angiogenesis for cancer development (Fig. 6).^119^

Adrenergic receptor (AR) activation by norepinephrine (NE) and/or E increases the expression of vascular endothelial growth factor (VEGF), matrix metalloproteinases (MMPs) through AR-dependent cAMP-PKA pathway and further promotes angiogenesis, proliferation and invasion.^120^ On the other hand, tumors drive neurogenesis to initiate new nerve fibers by the secretion of neurotrophic factors.^121^ This dynamic interaction constitutes a nerve-tumor-immune microenvironment, and the involvement of neural signals also increases the complexity of tumor microenvironment components.

#### T-cells

T-cells (Table 2) are the main components of adaptive immune system to kill infected cells and the major targets of cancer immunotherapy. The activation of adrenergic signaling from sympathetic nervous system halted mobility of immune cells and impaired T-cell responses through reducing local blood flow and limiting immune cells access.^109^ Pain-initiating sensory neurons release the neuropeptide CGRP to promote CD8^+^ T-cell exhaustion through CGRP-RAMP1 axis inhibiting their immune capacity to eliminate melanoma and enabling melanoma cells survival.^4^ The similar mechanism of midkine secretion acting on T-cells also exhibits in gliomas. NF1 gene mutations enhance neuronal excitability acting on CD8^+^ T-cells to secrete CCL4, leading to the secretion of a mitogen CCL5 from microglia, which elicits cell proliferation signaling in cell cycle.^122^ Ventrolateral medulla (VLM) catecholaminergic (CA) neurons promote tumor growth particularly mediated by regulating adaptive immune through CD8^+^ T-cells.^123^ Whether other immune cells, such as Treg cells and macrophages, are involved in CA neuron-regulated tumor growth mechanisms in other tumor types maintain exploration.

**Table 2:** T-cells alter neuronal signals to regulate cancer cell fate determination

T-cell	Neuronal signal that affects T-cells	Cancer type	Cancer cell fate
CD8^+^ T-cell	CGRP (exhaustion)	Melanoma cancer	Growth^4^
CD8^+^ T-cell	Acetylcholine (inhibition, at low CCL5 level)	PDAC	Growth^43^
CD8^+^ T-cell	Midkine (activation)	NF1 low-grade glioma	Growth^122^
CD8^+^ T-cell	VLM CA neurons (inhibition)	Colon adenocarcinoma	Growth^123^
CD8^+^ T-cell	α-MSH (inhibition)	Lewis lung carcinoma	Growth^125^
CD8^+^ T-cell	FMRP (inhibition)	PDAC, colon carcinoma, endometrial carcinoma, melanoma, and HNSCC	Survival^282^
CD8^+^ T-cell	Adrenergic signaling (exhaustion)	Melanoma and colon cancer	Accelerated growth^411^
CD8^+^ T-cell	GABA (inhibition)	Colon cancer	Survival^142^
Treg	FMRP (activation)	PDAC	Survival^282^
Treg	Dopamine (inhibition)	Unknown	Unknown^412^
Peripheral-Treg cell	Acetylcholine (inhibition)	Infectious diseases and cancer of the gut.	Increased susceptibility^413^
CD4^+^ T-cell	Cholinergic signaling (activation)	Hepatocellular carcinoma	Progression inhibition^414^

Neuroendocrine-immune system is integral to controlling cancer cell fate. Cancer cells release neurotransmitters/neurohormones and mediators into the circulation to activate receptors on neuron membrane or circulating immune cells to affect body homeostasis and tissue function in a mode favoring the tumor’s progression. For example, skin cancer-derived neurohormones, including corticotropin releasing hormone (CRH), and pro-opiomelanocortin (POMC)-related downstream hormones, act on other endocrine glands and immune cells affecting circadian rhythm, stress responses, tumor progression and immune escape via hypothalamic-pituitary-adrenal (HPA) axis. The processing of cancer neuroendocrine involves circulatory system, immune system and peripheral tissues by cortisol, biogenic amine (BA) and neuropeptides to generate a permissive microenvironment for the tumor’s phenotype and behavior alternation as well as react on nervous system disrupting brain function.^124^ Similar to the HPA axis, mice with different subcutaneous tumors exhibit hypothalamus activation and increased production of α-melanocyte-stimulating hormone (α-MSH) encoded by proopiomelanocortin (POMC) via the hypothalamic-pituitary (HP) unit to enhance tumor-induced myelopoiesis and immunosuppression by melanocortin receptor MC5R. Of these, tumor-derived α-MSH inhibits the tumor-infiltration of CD8^+^ T, CD4^+^ T and NK cells as well as the expression of interferon-γ (IFN-γ) in CD8^+^ T-cells to resist tumor immunity leading to tumor cell survival.^125^ T-cells are often known to exhibit anti-tumor immunity; however, tumor microenvironment can hamper this function by altering metabolic and epigenetic programs in T-cells.^126^ Understanding these complex interactions is essential for developing novel therapeutic strategies that can modulate the neuro-immune axis to enhance anti-tumor immunity.

#### Tumor-associated macrophages (TAMs)

TAMs, one of the most important tumor-promoting inflammatory cells, can be categorized into two types (M1 and M2). TAMs produce massive growth factors, small molecule cytokines, and MMPs that drive uncontrolled proliferation, promote EMT, support angiogenesis, aid the degradation of ECM, foster invasion, and suppress the adaptive immune system to help cancer avoid immune recognition and destruction.^127–129^ Tumor necrosis factor (TNF) is a chemoattractant for macrophages. Adrenergic nerves, which innervate spleen activated by vagal stimulation, promote splenic T-cells with β2-adrenergic receptors to secrete Ach that in turn inhibits the production of TNF of macrophages to suppress immune responses.^107,130^ Vagotomy can remove the immunosuppression and elevate TNF levels.^130^ Similarly, a phenomenon about the increment of nerve-dependent TAMs has been observed in breast, prostate, and pancreatic cancer.^131–133^ However, the sympathetic nervous system and parasympathetic nervous system appear to have the opposite effects on TAMs recruitment just like sympathetic nervous system signals to promote TNF release and TAM recruitment whereas parasympathetic nervous system might suppress these processes.^134^ TAMs represent one of the most abundant tumor-infiltrating immune cell types in the tumor microenvironment and present at all stages of cancer progression. Targeting TAMs has become a promising immunotherapy strategy, and understanding cancer neuroscience deeper may significantly improve the breadth and precision of this strategy.

#### Tumor-infiltrating B cells

Tumor-infiltrating B cells, including plasma cells and regulatory B cell (Breg cell) subsets, perform multiple functions in cancer treatment. Tumor-infiltrating B cells produce antibody and also participate in activating T-cell anti-tumor effect via unique pattern of antigen presentation.^135^ The development of B cell-based immunotherapeutic strategies via B cell activation performs effective antitumor responses which might be attractive in future clinic application.^136,137^ These B cell-based strategies are classified into activating cytotoxic B cells and/or inhibiting downstream immunosuppressive pathways, leading to antitumor immune responses.^138,139^ GABA is an important inhibitory neurotransmitter in CNS, and the GABA receptors have been clearly identified in multiple tumor tissues exerting regulative effects in cancer progression.^140,141^ B cells-derived GABA inhibits CD8^+^ T-cell killing function and enhances the recruitment of macrophages that secret IL-10, leading to anti-tumor immunosuppression mediated by the GABA receptor.^142^ Considering the regulatory role of B cells on neural signaling, it would be possible to forge B cells to adjust neuron function to remodel tumor microenvironments to ultimately inhibit cancer development.

#### Myeloid-derived suppressor cells (MDSCs)

Myeloid-derived suppressor cells (MDSCs) have the ability to significantly inhibit immune cell responses. Adrenergic signaling stress drives the immune suppressive activity of MDSCs mediated by β2 adrenergic receptor through decreasing glycolysis and increasing oxidative phosphorylation and FAO.^143^ Norepinephrine from adrenergic nerves also target to β2 adrenergic receptor on CD8^+^ T-cells and inhibit the expression of glucose transporter GLUT1 to suppress T-cells glycolysis and activation.^144^ Current link between neuro-tumor mostly through direct or indirect (via immune cells) effects of neurohormones/transmitters on cancer cells.^145^ It is highly agreed that the nervous system and peripheral organs exert metabolic regulation.

As described earlier (section “CGRP”), CGRP is released from nociceptor neurons, which directly induced the exhaustion of cytotoxic T-cells to impede their immune capacity to eliminate melanoma cells.^4^ Similar neural pathways have mentioned that stress-activated ventral tegmental area in brain regulates sympathetic innervation of the bone marrow, to alter the functional profile of MDSCs, and suppress melanoma and lung cancer growth.^146^

Recent studies have demonstrated that leukemia inhibitory factor (LIF) and Galectin-3 (Gal3) are elevated in the plasma of patients bearing different types of cancers, and these two cytokines can activate specific brain regions after being secreted by cancer cells.^147^ There may be a potential communication mechanism within the CNS in both peripheral cancers and intracranial tumors. Blockade of LIF and Gal3 or sympathetic nerves significantly inhibits MDSCs production and assume an antitumor immune response. The mechanism may involve peripheral cancer-initiated signaling connecting to the CNS. However, the causes of increased levels of LIF and Gal3 in peripheral cancers are still unclear. There may also be potential signaling molecules that help cancer cells sense the presence or absence of nerve and its signals in the microenvironment.

#### Cancer-associated fibroblasts (CAFs)

CAFs are a heterogeneous population of tumor stromal cells.^148^ In many types of tumors, dense extracellular matrix deposition inhibits immune cell infiltration through physical and chemical barrier action, creating an immunologically advantageous microenvironment. At the same time, the extracellular matrix components are changed into the form of type I collagen, which forms a super-large molecular polymer that promotes blood vessel growth and helps the migration of new blood vessels and nerves. CAFs can secrete and produce several growth factors, cytokines and/or chemokines, as well as exosomes to cross-link stromal cells and cancer cells.^149,150^ While increased extracellular matrix density early on helps prevent immune cells from entering, in advanced cancer, matrix metalloproteinases are secreted to degrade the extracellular matrix, allowing cancer cells to migrate and spread, known as collagen remodeling. Noradrenaline signaling acts on CAFs and promotes the type I collagen and secretion of MMPs to remodel the ECM and support tumor cell invasion.^151,152^ In pancreatic cancer, adrenaline signaling can enhance the expression of MMPs in stromal cells and promote the neural invasion of pancreatic cancer cells.^153^ This conclusion was also confirmed in the breast cancer model.^154^ Among them, beta-adrenergic blockers can save malignant progression. Schwann cells, one of the most important cell types in PNS, have important function in injured axons repair and new axon regeneration as well as tumor progression. Schwann cells activate the downstream STAT3 pathway to directly promote pancreatic ductal carcinoma cells perineural invasion through plasma membrane proteins (NCAM1) and secretory proteins (L1CAM and TGF-β). Schwann cells secrete chemokine to recruit various cell types via extensive communications with inflammatory macrophages fibroblasts and mast cells, promoting PNI and immune escape in PDAC.^155,156^ Schwann cells can drive tumor cells and CAFs in the pancreatic ductal adenocarcinoma microenvironment to achieve more malignant subtypes: basal-like and inflammatory CAFs, respectively.^155^ It means that refining Schwann cells to forge CAFs can be promising in obstructing the cancer cell proliferation and migration.

Collectively, in the field of tumor neuroscience, the immune microenvironment plays an indispensable role; and the research on neuro-immune direction is a heated area for tumor treatment, given the fact that it has more scientific supports than other fields discussed up to date.

### Inflammation

Chronic inflammation has been proved to be one of the main causes of tumorigenesis and malignant progression, which strongly modulates the body’s immune responses and leads to anti-tumor treatment immunosuppression.^157,158^ LCN2 is central to induce neuroinflammation, functioning in the innate immune system, which acts as part of the inflammatory process to combat bacterial infections. Signals secreted from the primary tumor into the blood stimulate the proinflammatory activation of astrocytes in the brain. Astrocytes promote the recruitment of myeloid cells from the bone marrow (granulocytes) to the brain, and they in turn become the primary source of LCN2 signaling. The level of LCN2 in the blood is closely related to an existing tumor, and high LCN2 levels signal a trend of brain metastasis and poor survival.^159^ Some anti-inflammatory drugs can significantly reduce the incidence of cancer and the risk of death, and some proinflammatory cytokines or stimulants can promote the infiltration of immune cells into infected tissues, thereby significantly improving the efficacy of cancer treatment.^160^ Non-specific anti-inflammatory drugs can inhibit chronic inflammation in the early stage, thereby hindering the occurrence of tumors and increasing the sensitivity of tumor cells to treatment. For certain specific inflammatory mediators produced during tumor development and treatment, targeted inhibition of inflammatory signaling pathways may be helpful to improve the effect of anti-cancer therapies such as chemo/radiotherapy and immunotherapy.

### Chronic stress

Chronic stress is widely considered one of the key promoters of cancer progression, especially in the context of neuro-immune-tumor interaction, which profoundly affects immune system function by activating the sympathetic nervous system and the hypothalamic-pituitary-adrenal axis, thereby promoting cancer occurrence, development and treatment resistance.

Chronic stress can induce stress-related hormones, catecholamines. Norepinephrine can stimulate the expression of hypoxia-inducible factor-1α, which is associated with VEGF secretion and tumor angiogenesis for metastasis.^161^ Using propranolol and galunisertib (Ly2157199) can block norepinephrine-stimulated cancer cell migration and invasion.^161^ In vivo, chemical consumption with 6-OHDA can reduce the release of catecholamines in tumor tissues, and inhibit the function of M2 macrophages, inhibit tumor growth by reducing tumor neovascularization. In vitro, catecholamine treatment can induce M2 polarization of macrophages and up-regulate VEGF expression, thus promoting tumor angiogenesis.^162^ At the same time, the adrenergic receptor antagonist propranolol can reverse the tumor-promoting effect of catecholamines under stress. A study uses an in-situ mouse model to simulate the complex interactions between pancreatic tumor cells and their microenvironment.^152^ in vivo optical imaging has been used to noninvasively track the growth and spread of primary pancreatic cancer. Stress-induced nerve activation increased the growth of the primary tumor and the spread of tumor cells to the normal adjacent pancreas, along with increased expression of invasive genes.^152^ At the same time, activation of β-adrenergic signaling induced a similar effect to chronic stress, and β-blocking reversed the effect of chronic stress on pancreatic cancer progression. These findings suggest that neural β-adrenergic signaling modulates pancreatic cancer progression under stress.

In addition, stress also increases the expression of MMP-2 and MMP-9 in tumor and stromal cells, prompting tumor cells to invade neighboring tissues.^152^ Recent work has shown that MMP-9 expression may drive neuronal damage in patients with colorectal cancer.^163^ In addition, in the inflammatory response of colorectal cancer, both the expression of MMP-9 and the expression of the pro-inflammatory factor IL-6 are upregulated, which may indicate that there are similar activation pathways in chronic stress and inflammatory response, leading to the spread of cancer cells and peripheral neuropathy, manifested by tumor metastasis and aggravated nerve pain.^163^ Chronic stress significantly increases the concentration of norepinephrine in serum and the expression of tyrosine hydroxylase in bone marrow. Norepinephrine activates the expression of pro-inflammatory factor IL-6, thereby activating the JAK/STAT3 signaling pathway, increasing the proportion of MDSCs in tumor tissue, and promoting breast cancer metastasis.^164^ β2-AR signaling modulates the expression of immunosuppressive molecules, such as arginase-I and PD-L1 and alters their ability to inhibit T-cell proliferation. The use of β2-AR antagonists can reduce MDSCs-induced immunosuppression.^165^

Chronic stress is also a predictor of recurrence in cancer patients. Norepinephrine can activate the ADRB2-CAMP-KA-CREB pathway to promote CLOCK transcription, leading to cancer stemness and resistance.^166^ Targeting this pathway, reducing the release of norepinephrine and inhibiting CLOCK expression, can counter the resistance of Kras^LSL-G12D/WT^ lung cancer to olanzapine, an antipsychotic drug.

Psychological depression, as one kind of chronic stress, has been shown to dysregulate the immune system and promote tumor progression.^133^ Neuropeptide Y released from norepinephrine-treated Myc-CaP cells promotes macrophage trafficking and IL-6 releasing, which activates STAT3 signaling pathway in prostate cancer cells, thus remodeling the prostate cancer microenvironment.^133^ Clinical specimens from patients with prostate cancer with higher score of depression revealed higher CD68^+^ TAM infiltration and stronger neuropeptide Y and IL-6 expression. These suggest that chronic stress is a contributing factor in cancer, but detrimental to survival.

## Therapeutic indication of cancer neuroscience

Discoveries in cancer neuroscience will optimize and even revolutionize oncological treatment.^6,167^ Targeting the interactions between the nervous system and cancer cells is most likely to become a supportive or alternative approach for oncological therapy, together with traditional therapeutic methods, including surgery, radiation, chemotherapy and immunotherapy.^168–170^ Neural signaling receptor or neurotrophin signaling pathways inhibition exhibits characteristics to either improve outcome and disease-free survival or provide immediate therapeutic benefit.^171–173^

Denervation strategies via blocking β-adrenergic receptors show potent effects in preventing metastasis.^8^ Surgical or chemical severing of sympathetic adrenergic nerve can inhibit the occurrence of prostate cancer, and blocking parasympathetic cholinergic nerve signal can reduce the spread of prostate cancer cells.^174^ However, different tumor tissues have different ways of innervation, such as secretory gland tumors (stomach, pancreas, and breast), and the degree of innervation is also different. Targeting neural input signals provides a promising approach for some specific tumors, but it also needs to be further explored in other tumors. For future efficient therapeutic exploration, it is crucial to decipher specific tumors-innervation crosstalk. However, a vital limitation of this approach lies on that the innervation of different tumor tissues may be specific, including the specificity of nerve species and the specificity of the degree of nerve signaling, as mentioned in a review written by Mancusi and Monje.^15^ In the era of targeted therapy, radiotherapy and chemotherapy combined with adjuvant treatment strategies, such as beta blocker, can provide a highly synergistic approach to controlling cancer progression. Considering the adverse effects of cancer in influencing the nervous system, such as seizures and nausea,^175–177^ improving the normal physiological activities of the body, such as sleep, anxiety and depression, and pain sensation, is also a valuable treatment choice.

It has long been suspected that biological behaviors, such as stress, depression, and social support, influence cancer development and disease progression, and this has indeed been demonstrated by research, and the molecular mechanisms of these effects are currently being explored.^161,178^ As we discussed above in the molecular mechanisms, recent findings in laboratory models suggest that biological behaviors can directly influence the functional activity of cancer cells through the neuroendocrine system. Stressful personalities, poor coping styles, negative emotional responses, and poor quality of life are associated with higher cancer incidence, poorer survival, and higher cancer mortality.

In order to visualize the existing therapeutic drugs, we summarized the current research status of neuro-drugs in anti-tumor in Table 3. Altogether, we believe that from the perspective of cancer, discussing the participation degree of cancer-related characteristics (gene expression patterns, morphological skeleton changes) in neuro-tumor interaction will help generate feasible research directions.

**Table 3:** Effects on cancer development by many representative neuronal signaling drugs

Neuronal signaling	Classification	Name	Effects on cancer (reference)
AchR	inhibitors	Vinblastine	Vinblastine destabilizes microtubule and drives macrophage polarization into the M1-like phenotype to exhibit antitumor immune effect.^415^
		Benzethonium Chloride	Benzethonium chloride can curb FGL1 secretion to disturb TAM-OTUD1-FGL1 axis and further inhibit liver metastatic tumor growth.^416^
		Otilonium Bromide	Otilonium Bromide inhibits USP28’s activity and causes cytotoxicity by down-regulating substrates c-Myc and/or ΔNp63, and enhance sensitivity to regorafenib in colorectal cancer.^417^
		Chelidonine	Chelidonine restricts the EMT and enhances antitumor effect of Lenvatinib in hepatocellular carcinoma cells.^418^
		Rocuronium bromide	Rocuronium bromide significantly weakens esophageal cancer progression, via the inhibition of autophagy and PI3K/AKT/mTOR signaling pathway in CAFs, to decrease the secretion of CXCL12.^419^
		Palmatine	Palmatine acting as one component of Zuojin capsule can target CDKN1A, Bcl2, E2F1, PRKCB, MYC, CDK2 and MMP9 to combat colorectal cancer.^420^
		Jatrorrhizine chloride	Jatrorrhizine inhibits mammary carcinoma cells by targeting TNIK mediated Wnt/β-catenin signaling pathway and EMT process.^421^
		Carvacrol	The combination therapy of carvacrol and sorafenib overcomes sorafenib resistance and cardiotoxicity in HCC by regulating TRPM7.^422^
		Carbaryl	The risk of thyroid cancer is inverse association with use of carbaryl.^423^
		SN-6	Acting as Na^+^ /Ca^2+^ exchanger (NCX) blocker, blocking the reverse NCX with SN-6 does not affect tumor cell viability.^424^ This case also happens in melanoma cells.^425^
	antagonist	Atropine	Atropine can effectively reduce EMT and colony formation induced by TGF-β or carboplatin in breast cancer cells.^426^
	agonist	Levetiracetam	Levetiracetam blocks the process that activated neuronal activity promotes microglial M2 polarization and GBM progression.^427^
	modulator	Ivermectin	Ivermectin decreases androgen receptor signaling and homologous recombination repair ability to resist prostate cancer.^428^
GABA receptor	activator	Methionine	Methionine secreted by tumor-associated vascular pericytes can promote tumorigenesis and tyrosine kinase inhibitors resistance.^429^
			Methionine deprivation induces irreversible cell cycle arrest and DNA damage in liver cancer cells by methionine catabolism pathway.^430^
	antagonist	Bicuculline	Bicuculline can prevent chemoradiotherapy-induced intestinal stem cells loss and intestinal damage but not decrease the chemoradiosensitivity of tumors, which is associated with L-type voltage-dependent Ca^2+^ channels.^431^
		4-hydroxybenzaldehyde	Various derivatives of 4-hydroxybenzaldehyde have anticancer effects.^432^
	agonist	Gabapentin	Gabapentin pharmacologically inhibits synaptogenic factor thrombospondin-1 to decrease glioblastoma proliferation by damaging the connectivity between glioblastoma and the normal brain.^62^
		Glabridin	Glabridin significantly down-regulates EMT and anti-apoptotic markers in cancer cells. And glabridin can delay paclitaxel metabolism and plasma exposure to enhance anti-metastatic effects in triple-negative breast cancer.^433^
		GABA	GPT2 overexpression activates GABA_A_ receptor by activating the delta subunit GABRD, and increases Ca^2+^ influx to promote breast cancer metastasis.^58^
	regulator	α-Pinene	Acting as OBP2A-binding ligand, α-Pinene significantly inhibits the function of OBP2A to suppress castration-resistant prostate cancer progression after androgen deprivation therapy.^434^
		Allopregnanolone	Allopregnanolone, steroid hormone progesterone (P4) and its neuroactive metabolite, promotes GB migration and invasion through c-Src activation.^435^
		Diazepam	Diazepam can induce cell cycle arrest in the G0/G1 phase, but diminish the anti-tumor effect in the combination with temozolomide.^436^
GluR	inhibitor	Evans blue	Evans blue modified FAPI-02 related radiopharmaceuticals significantly inhibit GBM cells growth with negligible side effects.^437^
	antagonists	MK-801 (NMDAR antagonist)	MK-801 blocks NMDAR, and further changes TAM phenotypes to promote anti-tumor immune responses.^438^
	agonist	L-glutamic acid	Poly-l-glutamic acid is conjugated to AVE1642, together enhancing the anti-tumor effect and inhibiting cancer cell proliferation/angiogenesis.^439^
Na^+^ channel	Na^+^ channel blockers	Lidocaine	Lidocaine, the most common used local anesthesia agents, exhibits anti-tumor effects.^440^

### DNA level

Some oncogenes can act as important components-mediated neural signaling functions. A remarkable result showed that the recurrence of glioblastoma is related to B-raf proto-oncogene (BRAF) kinase activation and Wnt/PCP pathway, showing a molecular transition to neuronal state.^106^ Inhibition of the proto-oncogene BRAF significantly inhibits the transformation and migration of neuronal features in recurrent tumor cells.^106^ We will discuss that there may be other proto-oncogenes/suppressor genes that regulate the module state transition to neurons (neurodevelopment, synaptogenesis, etc.) and altering their DNA levels may contribute to innovative cancer treatments.

#### Myc

**Fig. 7:**
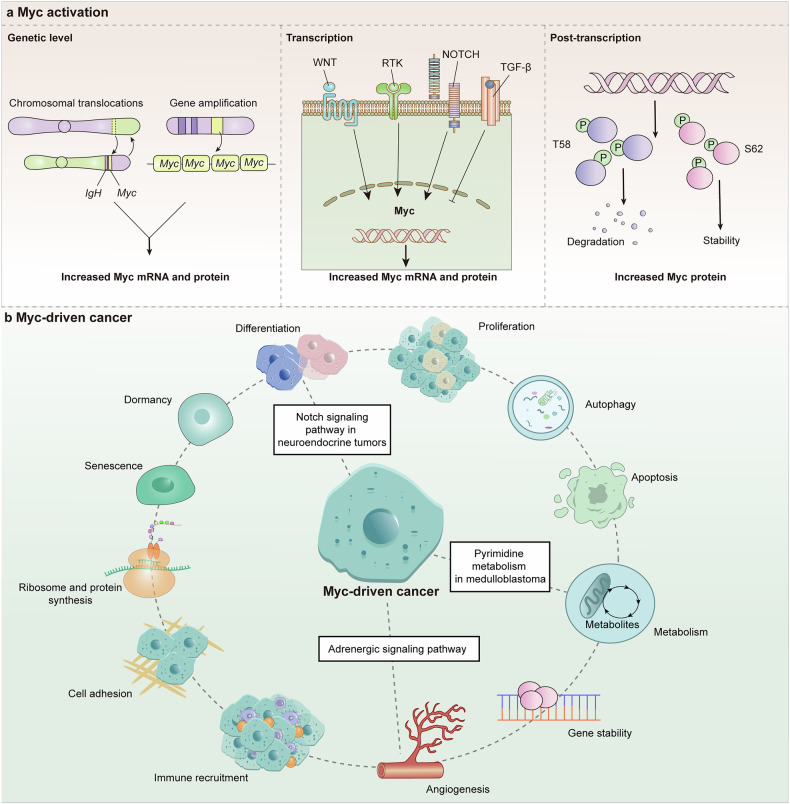
Myc-centered network in cancer neuroscience. **a** Myc activation in cancer at genetic, transcriptional, and post-transcriptional levels. Chromosomal translocations and genomic amplifications promote Myc expression. The alternation of upstream signaling pathways regulates Myc increased or decreased expression. Serine 62 (S62) preferential phosphorylation on Myc protein prevents Myc degradation from threonine 58 (T58) and promotes stabilization. **b** These hallmarks regulated by MYC work together to drive cancer malignant progression, participating in controlling proliferation, differentiation, metabolism, dormancy, senescence, protein and ribosomal biosynthesis, apoptosis, autophagy, and so on (The arrows represent induce, and the horizontal lines represent inhibit)

Myc is one of the well-characterized proto-oncogenes encoding nuclear transcription factors and includes three types: c-Myc, n-Myc, and l-Myc.^179^ Myc mediates multiple biological programs primarily as a transcription factor regulating 1000 gene expression (Fig. 7).^180–182^

Myc has oncogenic effect on nervous system-derived tumors, such as neuroblastoma and GBM,^183,184^ whose effects are not limited to these tumors but involved in development and repair of neurons and glial cells. In the context of nerve injury, specific overexpression of c-Myc in microglia induces microglial proliferation and neuropathic pain.^185^ In the injured retinal ganglion cells and peripheral nerve, both mRNA and protein levels of c-Myc are transiently upregulated to promote axon and nerve regeneration.^186,187^ From these data, c-Myc may have the potential for regulating neuroinflammatory homeostasis and repair, not just limited to cancer progression.

c-Myc acts as a component of Yamanaka factors can induce somatic cell reprogramming into iPSCs, and also n-Myc and l-Myc.^188,189^ Inducing differentiated mature or aged neurons back to a younger state through cellular state reprogramming is feasible, and the factors are also pluripotent and direct reprogramming factors, including c-Myc.^190^ Hence, Myc can act as a bridge for linking nervous system to cancer. Myc drives the alternation of small cell lung cancer subtypes progression along a single evolutionary trajectory by activating the Notch signaling pathway and then inducing neuroendocrine dedifferentiation.^191^ In orthotopic xenograft and Myc-driven genetic mouse models of prostate cancer, the expression of β2-adrenergic receptors of adrenergic signaling pathway on tumor stroma endothelial cells decreases oxidative phosphorylation, thus promoting angiogenesis in tumor progression.^44^ In a Myc-driven prostate cancer mouse model, doublecortin (DCX^+^) neural progenitors infiltrate and reside in prostate tumor tissue. These DCX^+^ neural progenitors subsequently generate new adrenergic neurons to promote tumor growth and metastasis.^11^ The density of DCX^+^ neural progenitors is strongly associated with the aggressiveness and recurrence of prostate adenocarcinoma. These findings underscore the multifaceted roles of CNS in both cancer biology and neuroscience, positioning oncogene-driven tumor and nervous system as a key player in the interface between these two fields.

Myc signaling targeting may be a potential therapeutic approach for cancers, but its trials are not quite successful due to the loose structure of MYC protein lacking suitable domain pockets for binding.^192^ Myc also drives embryonic development, tissue repair and chromosome remodeling in the nucleus, while abnormal regulation of Myc can promote malignant transformation and reprogram adult cells to a “reset” stem cell state.^193–195^ This makes targeting Myc extremely difficult due to its important noncancerous regulatory function in normal cells and tissues.

#### Ras

Ras is the most frequently mutated oncogene family found in numerous cancer types, with three isoforms K-Ras, N-Ras, and H-Ras.^196,197^ More recent studies revealed new druggable sites in the switch-II pocket of KRAS, offering a new potential direction for the development of inhibitors by directly binding to Ras.^198,199^ The overactivation of the RAS-MEK pathway can drive various clinical manifestations of the genetic syndrome neurofibromatosis type 1 (NF1).^200^ Blocking the downstream signaling pathway of Ras by MEK inhibitors has been shown to reduce the size of NF1-related nerve-derived tumors, such as neurofibromatosis, plexiform neurofibromas, and malignant peripheral nerve sheath tumor.^201^ Similarly, other inhibitors of downstream of Ras, such as mTOR inhibitors, have both the anti-tumor effects in tumor progression and emotional and cognitive improvement on hippocampal neurons (such as reducing depressive-like behavior) without causing tumor regression.^202^ These information suggests that mTOR inhibitors play different roles in tumors and nerves, highlighting the complexity of targeting Ras signaling pathways. Therefore, it may be difficult to find a drug that can act on both peripheral and intracranial tumors from peripheral chemotherapy drugs.

The Ras family also possesses a precise control during the whole stages of axonogenesis. This involves the dynamic alternation of actin and microtubule cytoskeletons, the interaction between growing axon and cells or extracellular matrix, and the transport of related protein.^203^ Oligodendrocyte myelination through multiple signaling pathways, such as PI3K/Akt/mTOR, Erk1/2-MAPK, and Wnt/β-Catenin, is responsible for protecting axons.^204^ Members of the Ras family, such as R-Ras1 and R-Ras2, are revealed as intermediaries to link myelination pathways.^204,205^ The above studies suggest that Ras may regulate the generation or differentiation of neuro-tumor synapses.

#### FMS

FMS (known as colony-stimulating factor receptor, CSFR, CSF-1R), which is activated by its ligands, CSF-1 and IL-34, is a type III tyrosine kinase receptor as a membrane-bound enzyme, which was initially described as an oncogene.^206^ FMS participates in myelinogenesis via regulating the activation of microglia, which are the most important immune cells in CNS.^207^ Under the conditions of NMDA-induced neurotoxicity and teratogen cyclophosphamide, increased expression of FMS causes microglia activation, cytokines release, enhanced phagocytosis, and migration to injured neuronal cultures, reducing neurotoxicity and teratogen-induced injury, preventing neurons from injury.^207^ Using FAM inhibitor can lead to microglia/macrophage depletion and repopulation to reduce neurodegeneration and foster synapse recovery after traumatic brain injury.^208^ Similar to the results discussed in c-Myc, neuroinflammation after nerve/brain injury shows changes in oncogene expression, which in turn regulates the activation and migration of microglia, and synaptic production. Glioma cells tend to migrate toward glioma-associated microglia/macrophages-conditioned media activated by GM-CSF in which CCL5 (inflammatory mediator produced by immune cells) was abundant.^209^ Most evidence supports that FMS (CSFR) -CSF axis is involved in tumor development and treatment, as well as neural development and repair pathways in the form of intermediates. It can also be seen that in the current summary of *Myc* and *FMS* oncogenes, neuroinflammation with microglia activation has a wide range of oncogenes regulating synaptic production, either as initial regulation or in the form of activation intermediates. This observation prompts further consideration of the mechanisms underlying neuro-tumor connection.

#### HER2

The β-adrenergic signaling pathway stimulates a variety of cancer-causing signaling pathways, including those of Src and HER2 (encoded by ERBB2). In HER^+^ cells, the β2-adrenergic receptor and HER2 form a positive feedback loop in human breast cancer cells, and the β-adrenergic receptor activates the signal transductor and transcriptional activator 3 (STAT3), which in turn activates the ERBB2 promoter to stimulate gene transcription.^210^ Catecholamines effectively antagonize the anti-proliferation effect of trastuzumab both in vitro and in vivo. Trastuzumab-resistant dependent PI3K/Akt/mTOR pathway is controlled by catecholamine-induced β2 adrenergic receptor activation. The β-blocker propranolol not only enhances the anti-tumor activity of trastuzumab, but also re-sensitizes resistant cells to trastuzumab.^210^

Moreover, β-adrenergic receptors mediate norepinephrine to induce SRC-S17 phosphorylation via the ADRB/cAMP/PKA axis and further activate Y419 phosphorylation, leading to tumor growth in breast. In human ovarian cancer samples, high tumor norepinephrine levels are associated with high levels of pSrc^Y419^.^211^ Catecholamines stimulate the activation of ADAM10 expression and proteolytic activity, and catecholamines induce the activation of gamma-secretase, resulting in abscission of ADAM10 from the HER2 extracellular domain and subsequent intramembrane cleavage of the HER2 intracellular domain by presenilin-dependent gamma-secretase. Nuclear translocation of the HER2 intramembrane domain and transcriptional enhancement of COX-2, a gene associated with tumor metastasis. Nuclear localization of HER2 is significantly associated with overexpression of β2-adrenergic receptor in human breast cancer tissues, which plays a decisive role in tumor metastasis.^212^

Of these, ADAM10, a transmembrane zinc-dependent protease, has the function of proteolysis and can cleave a variety of transmembrane proteins (such as Notch, E-cadherin, HER2, VEGF, etc.) to regulate tumor-related signaling pathways. The high expression of ADAM10 is closely related to the enhancement of breast cancer metastasis and invasion ability and the decrease in the survival rate of patients.^213^ To target ADAM10, inhibition of HER2 cleavage by the ADAM inhibitor INCB7839 may enhance the clinical efficacy of trastuzumab in HER2^+^ breast cancer patients.^214^ As discussed above, ADAM10 promotes glioma cell proliferation and drug resistance.^1^ In addition, ADAM10 is screened as a target for adavivint (SM04690) to specifically regulate NOTCH2 protein expression, which mediates transcriptional regulation of the Wnt pathway. NOTCH2 directly activates transcription of TCF7L2 and Wnt target genes, such as MYC, JUN, and CCND1/2, rather than the downstream transcription factor critical for classical Wnt/β-catenin signal transduction, such as TCF7-like 2 (TCF7L2).^215^ ADAM10 inhibitor, GI254023X,^216^ can reverse anti-PD-1 resistance in human tumor-infiltrating lymphocytes, which necessitates effective clinical treatment.

#### Rb

Rb is the first identified tumor suppressor gene in retinoblastoma capable of regulating cell cycle, growth, and metabolism. Retinoblastomas are a cancer highly likely initiated by Rb mutation. A study has shown that mouse retinoblastomas has surprisingly high neuronal differentiation in a series of knockout mouse models.^217^ In these models, early-stage mouse retinoblastoma cells can form synapses and extending neurites, which exhibit similarity as amacrine or horizontal cells of the retina. However, as the disease progresses to the late stage, mouse retinoblastoma cells lose neuronal differentiated morphology, and become more invasive and aggressive. During the transition from early-stage to late-stage retinoblastoma cells, neurites and synapses can construct extensive cell-cell contacts.^217^ The validation consistent with the results of high neural involvement is that Rb protein was early found to widely express in adult sensory neurons and axons, and its downregulation can promote neuronal regeneration, including neurite growth and branch formation.^218^ Fully differentiated horizontal interneurons with neurites and synapses can reenter the cell cycle and proliferate to form cancer, while maintaining their differentiated state.^219^ One characteristic of retinoblastoma is the infiltration of optic nerve.^220,221^ These facts highlight the close connection between the loss-of-Rb-driven retinoblastoma and nervous system.

#### NF1

NF1 acts as a tumor suppressor gene, and its mutations are associated with the initiation of optic pathway gliomas derived from optic nerve glial cells.^25^ As mentioned above, NF1 mutations enhance the aberrant shedding of neuroligin 3 (NLGN3) from the postsynaptic membrane, to promote optic pathway glioma growth. The inhibition of NLGN3 shedding by knockdown or using sheddase inhibitor can impede NLGN3 production and subsequently decrease tumor growth.^25^ In NF1 mutation-driven optic pathway glioma mouse models, stimulating optic nerve activity can augment optic glioma growth, while reducing optic experience, such as light deprivation, can prevent tumor formation and maintenance.^25^ This raises a question of whether other sensory experiences besides visual experience, such as smell and taste experience, also have an impact on tumors within the corresponding neural pathways. Olfaction has been shown to directly regulate gliomagenesis.^40^ They all suggest that elements of functional sensory experience input circuits are key targets for potential treatment of tumor neuroregulation.

Neurofibroma type 1 can arise from any part of the visual system, including the optic nerve, optic chiasm, optic tract, optic radiation, and hypothalamus. Among them, as photoreceptors, rod cells are the first level neurons in the retina, which convert light into electrical signals. Retinal neurons transmit signals to the brain to form visual experience. NF1 mutations are associated with rod cell dysfunction, but there is no clear scientific evidence of a direct link between rod cells and optic glioma, so it cannot be considered as causal relationship. However, as a part of the eye, rod cells can be affected by optic neuropathy. If the optic nerve is compressed or invaded by the tumor, it may influence the function of the rod cells and thus affect the vision. Notably, one previous study revealed that NF1 deficiency can enhance cell proliferation while limit oxidative ATP production and energy flexibility.^222^ This phenomenon implies that NF1 mutations may indirectly influence rod energy metabolism. Mechanistic targets may also be explored to find link between metabolic properties of rod hypoxia to NF1 mutations. During state transition and totipotency acquisition in pluripotent stem cells, mitochondrial TCA cycle enzymes are translocated to the nucleus.^223^ If the translocation of mitochondrial TCA cycle enzymes to the nucleus occurs during rod development, this may provide a new perspective on the “NF1 mutation-rod metabolism-NLGN3 shedding” link. Although the study as mentioned above has shown the neuron development associated with GBM progression, there is limited evidence for the connection of lactate, axon biology, and brain tumor cells, which might be an emerging opportunity for exploring cancer target treatment.

#### p53

Various targeting therapeutic strategies in response to p53 expression levels have been used to regulate cancer cell cycle, energy metabolism, and autophagy, thus prohibiting tumor progression.^224–227^ RNA sequencing analysis of exosomal miRNAs shows that differentially expressed genes are highly correlated with neuronal growth, morphogenesis, synaptic formation, differentiation, dryness, and synaptic transmission. The expression of miR-34a and miR-141-5p in the exosomes of p53-deficient oropharyngeal carcinoma is significantly down-regulated, while inhibition of miR-34a and miR-141-5p in the exosomes of wild-type p53 cells could produce a p53-like effect.^12^ However, the effect of miR-141-5p is weak, suggesting that miR-34a and miR-141-5p in exosomes are involved in neural reprogramming, and miR-34a played a major role. In oral cavity squamous cell carcinoma, loss of p53 results in the secretion of cancer-derived extracellular vesicle (known as exosome) without miR-34a, which not only drives adrenergic neuritogenesis but also induces sensory nerve reprogramming into adrenergic neuron, and the adrenergic neuron-derived signals further promoting cancer cell growth.^12,228^ In vivo studies have shown that adrenalin secretion can be detected after injection of p53-deficient exosomes into tumors, suggesting that p53-deficient exosomes can induce neural reprogramming, and that the knock-out of Rab27A and Rab27B can inhibit this process. Rab27A and Rab27B are key proteins released by extracellular vesicles and are involved in intercellular communication and tumor microenvironment regulation. Interfering with these proteins can impair vesicle transport and selective payload, inhibiting cancer phenotype deterioration. The communication between neurons and p53 is involved in regulating synaptic plasticity, axon outgrowth, and neuron development.^229^ In addition, chronic stress- epinephrine decreased the stability of p53 protein by promoting p53 ubiquitination. It is suggested that the increase of sympathetic nervous system activity may promote tumorigenesis or chromosomal instability.^230^ Long-term activation of adrenaline signaling leads to ARRB1/AKT-mediated activation of MDM2, an E3 ligase, thus driving DNA damage and inhibiting p53 level.^231^ It is worth noting that nerve distribution varies significantly according to the physiological conditions of different tissues and cells, and the distribution of adrenergic receptors in different cancer cells also has its own characteristics. Considering the facts that current cancer treatment of targeting p53 is through stabilizing or preventing the degradation of p53 protein via acetylation, de-ubiquitination, and/or non-coding RNA, exploring the neurotransmitters and certain electrochemical signaling to regulate p53 stability and further inducing the death of neuron which is hijacked by cancer cell to inhibit tumor growth might be a direction for cancer treatment.

#### DNA therapeutics

Current understanding suggests that the mutation or loss of several tumor suppressor genes regulated by neural signals can influence neuron to glioma synapses, assuming that even no breakthrough can be made in inhibiting the malignant progression of tumors, researchers can look into its important regulatory role in development and differentiation. The combination of drugs targeting oncogene signaling with neural regulators may have potential applications in cancer treatment.

Using the CRISPR/Cas system, one can precisely modify the genomes of cancer cells and neurons, which helps to study specific signaling pathways as a current laboratory routine.^232,233^ In particular, CRISPR allows researchers to develop more accurate models of cancer and explore how tumors interact with the nervous system. For example, studies have found that certain neurotransmitter receptors or genes associated with tumor growth are mutated, which can cause tumors to become resistant to treatment. Given Cas9-induced safety risks, researchers need to pay special attention to long-term safety of gene therapy in the body.^232^

Notably, the study of intracranial tumors based on genomics and epigenomics should pay attention to the specificity of tumor type, and potential relationship between neuron origin and malignant molecules.^49^ The complexity of interactions between cancer cells and nervous system is the most significant challenge. The technological advances and innovations in genomics are mind-blowing.^234^ By combining information about neuronal signature proteins, it is possible to gain neuroregulatory preferences in pre-cancer, metastatic, and advanced stages, which will help us find ways to crack tumor metastasis and drug resistance. In addition, certain cancer types may have specific gene expression during metastasis, which has been demonstrated in several studies.^235,236^ Blood and cerebrospinal fluid (CSF) are the most commonly used biological fluids to collect circulating cells and biomolecules of tumor origin. There is great potential that in combination with cerebrospinal fluid biopsies and single-cell gene sequencing will help more quickly obtain markers of relevant neuro-tumor communication, which may contain some cancer therapeutic targets through gene regulation or editing.

### RNA level

In addition to genetic sequence mutations, it is crucial to pay close attention to epigenetic modifications that contribute to regulating carcinogenesis.^237^ The new finding identifies regulating bromodomain-containing protein 8 that occupies p53 target loci by enforcing a compact chromatin conformation to prevent p53 activation and promote GBM proliferation in a GBM-specific epigenetic mechanism.^238^ Epigenetic modifications, including those mediated by RNA-binding proteins (RBPs), noncoding RNAs (ncRNAs), and RNA modifications, play a crucial role in regulating gene expression and determining tumor cell fate, making them significant contributors to carcinogenesis. The epitranscriptome, an emerging component associated with human diseases, embeds RNA modifications that regulate RNA molecule activity. The RNA modifications have been proven to present in multiple RNA types, including coding RNA and non-coding RNA, all of which tightly link to carcinogenesis.^239^ A few reports have described the relationship between neural cells and tumor cells at RNA level during cancer malignant progression by single-cell RNA-seq.^240,241^ On the one hand, the developmental origin of ependymoma, a central system tumor, has been linked to neuronal-glial fate;^240^ and on the other hand, chemosensitivity of peripheral tumor cells and expression of ion signaling channels, are neuron-sensing Ca^2+^-influx channels.^241^ It is reasonable to understand that cell differentiation, axon formation, and ion transport channels in the nervous system may be important parts of the discussion of cancer neuroscience. There are great mysteries in the transcriptome, which serves as a worthwhile junction between cancer and neuroscience. We would discuss this in more detail below with a potential link for RNA-based therapy of tumors.

#### mRNAs and noncoding RNAs

Increasing studies have proven that aberrant mRNA translation widely participates in cancer initiation and progression.^242–244^ The treatment of tumors at the mRNA level is mostly immune direction, which control the expression of immune checkpoint molecules by the precise regulation of mRNA translation, to modulate the generation and presentation of aberrant peptides antigen, and affect the functional differentiation of tumor-associated immune cells.^245–251^ The toxicity and immune-related side effects of immune checkpoint inhibitors (ICI) can be seen in any organs, including the nervous system, providing the link between RNA and immunity to neuronal cancer. Toxic side effects on the nervous system include meningitis, neuroinflammation (peripheral nervous system myositis), and peripheral neuropathy.^252,253^ Alternation of the expression of neural signaling molecules that we discussed above in Section 2 would have a promoting effect on the cancer cells’ progression. Although they may directly function in the protein form, such as BDNF, NLGN3, β-adrenergic receptor, etc., they may produce similar effects through their counterparts if they can be inhibited at the mRNA stage, thereby inhibiting neural-tumor communication. However, our discussion is very limited by the fact that the major studies have not yet addressed the mechanism at the RNA stage. The histone methylation of BDNF is regulated by lysine demethylase 5C (KDM5C).^254,255^ In Alzheimer’s disease, KDM5C binds to a repressive element in the BDNF promoter region and reduces the nearby histone H3 lysine 4 di- and tri-methyl modification, thereby suppressing BDNF transcription.^254^

Only less than 2% of the human gene sequences can code proteins, while 90% are transcribed into RNA.^256^ Non-coding RNAs do not encode proteins but play essential roles in a variety of processes, including modulation of gene expression and responses to cellular pathway.^257,258^

MicroRNAs (miRNAs), a class of non-coding RNA molecules with a length of about 200 nt, regulate gene expression at the post-transcriptional level and participate in multiple biological processes (Table 4).^259^ miRNAs play an unprecedented role in nervous system development in which they can impact neuronal regeneration. TDP-43 is an RNA/DNA binding protein, which is critical for the development of neurodegeneration, and a number of TDP-43-associated miRNAs are involved in the progression of human cancers, such as lung cancer and triple-negative breast cancer.^260,261^ miR-34a in exosomes from p53 wild type cells inhibits the alternation of neuron and nerve reprogramming which further inhibits oral cavity squamous cell carcinoma cells survival.^12^ In brain tumor, miR-155 coated by virus-mimicking nucleic acid nanogel can reprogram microglia and macrophages that are infiltrated in GBM tumor microenvironment from tumor-supporting into anti-tumor phenotype and consequently inhibit tumor growth.^262^ These results prompt us to speculate that exploring miRNA regulation of neuronal differentiation, synapse formation and neurite generation might provide new therapeutic opportunities for intracranial tumors, such as targeting neuronal tumor synaptic marker molecules or tumor cells by designing exosomes coater by the membrane of microglia.

**Table 4:** Representative non-coding RNAs that help modulate cancer cell fate determination

Non-coding RNAs	Representative function in cancer cell fate	Representative function in neuroscience	Clinical trials
miR-21	Downregulates the expression of tumor suppressor gene PTEN by increasing methylation level on PTEN promoter region to promote cancer cell proliferation and invasion^441^	Leads to local inflammation and neuropathic pain after peripheral axonal injury;^442^ repairs spinal cord injury;^443^	lack of relevant data
miR-19a	Binds to 3’-UTR of T-cell intracellular antigen 1 (TIA1, a tumor suppressor) mRNA and downregulates TIA1 expression to promote cancer cell growth and migration^444^	Promotes axon regeneration and extension by miR-19a-PTEN axis in optic neuropathies^445^	lack of relevant data
miR-373	Inhibits the YAP1 repressor LATS2, and together with EMT-activating transcription factors ZEB1 to upregulate the expression of YAP1, thus promote the production of integrins α3 (ITGA3) to promote cancer cell metastasis^446^	Participates in AD pathogenesis^447^	lack of relevant data
miR-27b-3p	Downregulates the expression of vascular endothelial cadherin and p120 at post-transcriptional level, and also downregulates gap junction protein GJA1 expression under hypoxia to promote cancer cell metastasis,^448^ and growth^449^	Associates with bipolar illness and schizophrenia;^450^ regulates synaptic development mediated by Bmi1^451^	lack of relevant data
miR-519a	Inhibits STAT3/Bcl-2/Beclin-1 signaling pathway to promote cancer cell autophagy and apoptosis^452^	Elevates MPP^+^ neurotoxicity in dopaminergic SK-N-SH cells by sponging miR-519a-3p via ceRNAs (SNHG14 and ATG10)^453^	lack of relevant data
	Activates the MAPK/ERK pathway by targeting DUSP2, and causes M2-like polarization of macrophages to promote liver metastasis^454^		
miR-124	Downregulates STAT3 to promote cancer cell proliferation inhibition^455^	Repairs nerve injuries;^456^ participates in multiple neurodegeneration disorders;^457–459^ links the polygenic risks to behavioral changes in psychotic disorders (schizophrenia and bipolar disorder)^460^	ABX464 (obefazimod), upregulating miR-124 in immune cells, used in the treatment for patients with moderate-to-severe, active ulcerative colitis^461^
miR-182	Suppresses TLR4 to result in NF-κb inactivation and M2 polarization of TAMs to promote cancer cell growth^462^	Increases neurite outgrowth and mediates neuroprotection of DA neurons in PD;^463^ Inhibits the expression of Rac1 of injured neurons to inhibit neuroinflammation and improved brain injury after ischemic stroke^464^	The expression of the miR-182 cluster upregulates the possibility of CD4 + FOXP3 + IL-17-producing Treg formation in breast cancer from meta-analysis^465^
	Exosomal miR-182/183-5p in bile targets hydroxyprostaglandin dehydrogenase in CCA cells and MCs to promote cholangiocarcinoma self-driven progression^466^		
miR-146a-5p	Secreted and transferred to astrocytes via EV delivery and inhibits NUMB in the Notch signaling pathway to promote brain metastases^467^	Secreted by microglia to suppress neurogenesis and spontaneous discharge of excitatory neurons in depression by targeting KLF4^468^	miR-146a-5p can be a good candidate for identification as predictive markers of predictive biomarkers of duloxetine (a serotonin-norepinephrine reuptake inhibiter) response in major depressive disorder patients^469^
miR-99a	miRNA microarray analysis in HCC tumor liver tissue and non-tumor liver tissue determined that let-7c, miR-199a-3p and miR-99a were down-regulated in tumor tissue. The X antigen HBx encoded by hepatitis B virus enhances EZH2 expression and attenuates the expression of Mir-Let-7C to induce HMGA2 expression in HCC cells. Knockdown of HMGA2 significantly down-regulates HBX-induced metastasis potential of HCC cells^470^	Hypothalamic miR-99a levels are significantly decreased, and its target FK506-binding protein 51 is significantly increased. And synaptic proteins, including postsynaptic dense protein 95 and synaptophysin, show similar reductions in the hypothalamus in a novel peri- to postmenopausal depression model induced by a two-step ovariectomy plus chronic mild stress^471^	The antipsychotic drug aripiprazole suppresses colorectal cancer by targeting LAMP2a to induce RNH1/miR-99a/mTOR-mediated autophagy and apoptosis^472^
lncRNA HOTAIR	Interacts with membranes of multivesicular bodies (MVBs) and Ras-related protein Rab-35 expression, as well as the colocalization of vesicle-associated membrane protein 3 (VAMP3) and synaptosome-associated protein 23 (SNAP23) to promote cancer cell progression^473^	Increases MPP^+^-induced neuronal injury in SK-N-SH cells by modulating the miR-874-5p/ATG10 axis^474^	lack of relevant data
lncRNA MALAT1	Forms a complex with c-MYC to combine with the promoter region of KTN1 gene, thus promote EGFR protein expression to promote cancer cell proliferation and metastasis^475^	Improve spinal cord injury via the miRNA-22-3p/SIRT1/AMPK axis;^476^ participates in rod photoreceptor degeneration^477^	The most robust correlation among patients was detected between ANRIL and MALAT1 lncRNAs in acute and chronic inflammatory demyelinating polyneuropathy, which can be regarded as a sensitive and specific diagnostic panel for acquired immune-mediated polyneuropathies^478^
	Increases TCF7L2 translation mediating elevating glycolysis and reducing gluconeogenesis to promote cancer cell progression^479^		
lncRNA SNHG12	Activated by DNA methylation binds to miRNA to regulate MAPK/ERK pathway and G1/S cell cycle, and further acquires TMZ resistance to promote cancer cell temozolomide resistance^480^	lack of relevant data	lack of relevant data
lncRNA-H19	Slience of lncRNA-H19 exhibites significantly superior therapeutic efficacy in preventing primary tumor growth and lung metastasis, in orthotopic and lung metastasis mouse models of colorectal cancer^481^	Increased expression of lncRNA-H19 significantly promotes NLRP3/6 inflammasome imbalance and resulted in microglial pyroptosis, cytokines overproduction, and neuronal death, by forming ceRNA network with miR-21^482^	Genomic variants within the lnc-RNA H19, including the T C haplotype (rs2839698 and rs217727) and the C T haplotype, has different preferred risk of breast cancer in Iranian population^483^
lncRNA NEAT1	High m6A level of NEAT1-1 associates with bone metastasis of prostate cancer by controlling complex CYCLINL1/CDK19/NEAT1-1 formation and Pol II ser2 phosphorylation^484^	As the scaffold of nuclear paraspeckles, upregulation in stressed neurons promotes TDP-43 liquid-liquid phase separation, acting as a stress-mitigating role in ALS pathogenesis^485^	lack of relevant data
	Upregulated in cancer patients and increases high and efficient glycolysis by binding and forming a scaffold bridge for the assembly of PGK1/PGAM1/ENO1 complexes, to further promote breast cancer growth and metastasis^486^		
lncRNA MEG3	Shows anti-tumor properties (osteosarcoma (OS)) by combining exosomes and lncRNA MEG3^487^	Up-regulated in AD patients and induce necroptosis in human neurons^488^	Acting as tumor suppressor, lncRNA MEG3 polymorphisms are associated with the response and toxicity of chemotherapy drugs, such as paclitaxel and cisplatin^489^
	Activates NLRP3/caspase-1/GSDMD pyroptosis pathway in triple-negative breast cancer^490^		

It is also worth elaborating on the types and splicing patterns of miRNAs in intracranial tumors, such as whether there are homologous sequences or similar splicing patterns between oncogenic/cancer-promoting miRNAs and neuronal differentiation-promoting miRNAs. Microglia and macrophages accumulate in GBM but not induce anti-tumor inflammation reactions.^263^ Of these, the anti-tumor capability of microglia is inhibited by epigenetic silencing with histone modifications. Considering the PNI characteristics of tumor cells, a previous review has drawn a conclusion of non-coding RNAs playing a major role in injured peripheral nerve repair.^264^ Given that the synapses can be mediated by NMDAR between breast-to-brain cancer and neurons, miRNA might drive breast cancer to undergo brain metastasis. However, whether different species or expression levels of miRNAs regulate the whole process of metastasis, and how the generation of synapses varies at different stages of metastasis require further exploitation.

Long non-coding RNA (lncRNA) with more than 200 nt as length, plays a major role in tumor-supporting and -suppressive effects^265–267^ (Table 4). Mechanistically, the regulatory locus of p53-induced lncRNA-p21 transcription activating the cis-regulation depends on the conserved regions in exon 1 of lncRNA-p21.^268^ lncRNAs can also direct protein binding partners to regulate gene expression to modulate neuronal development and function. lncRNAs control neuronal development via m6A modification and axon elongation via regulatory protein.^269^ Axon-enriched lincRNA ALAE binding with KHSRP can maintain Gap43 mRNA translation to locally enhance axon elongation of dorsal root ganglion neurons.^270^ This function of regulating synapse and axon elongation by lncRNA is important for neuro-glioma synapse formation and communication.

#### RNA-binding proteins

RNA-binding proteins and RNA modification involved in RNA splicing and translation are particularly relevant in neurogenesis and neuronal function as well as nervous system development.^271,272^ IGF2BP proteins, including IGF2BP1, -2, and -3, are well-known RNA-binding proteins that embody markedly important function in distinct aspects of oncogene expression associated with carcinogenesis, cell cycle, metastasis, metabolism, drug resistance, and relapse.^273–275^ IGF2BP protein is involved in the malignant process of cancer^276–278^ and also plays an important regulatory role in the nervous system. IGF2BP proteins act as key regulators in neuronal development, including synaptic terminal growth, neural cells differentiation and migration, neurogenic potential alternation, axons growth, dendritic branching, and nerve regeneration.^279^

Splicing factor polypyrimidine tract-binding protein 1 (PTBP1), acting as an RBP together with paralog PTBP2, controls the event of neural differentiation. The splicing co-factor SON that has the highest expression level in GBM can upregulate PTBP1-mediated oncogenic transcript splicing and suppress RBFOX2-mediated PTBP2 pro-neuronal splicing by forming a complex with hnRNP A2B1 in GBM.^280^ The RBP-mediated program markedly reduces GBM cell growth and eradicates the stemness of glioblastoma stem cells, while SON knockdown can significantly inhibit GBM cells growth as well as suppress intracranial tumor growth.^280^ Fragile X mental retardation protein (FMRP), a neuronal RNA-binding protein, controls synaptic plasticity in neurons via glutamate-stimulated NMDAR signaling and broadly governs protein translation and mRNA stability in multiple human solid tumors.^5,281^ The expression of FMRP promotes the secretion of cytokines to induce Treg cells and represses chemoattractant C-C motif chemokine ligand 7 to prevent CD8^+^ T-cells attack, ultimately achieving cancer immunosuppressive effects.^282^ High expression of RNA-binding protein SERBP1 is prevalent in GBMs, which correlates with poor survival of the cancer patients. SERBP1 regulates serine and one-carbon unit cycle metabolism to control methionine production, which is associated with genome methylation, further inhibiting the expression of genes associated with neurogenesis, synaptogenesis, and neuronal differentiation while promotes GBM stem cell phenotypes and radiation resistance. The function of SERBP1 that acts as a bridge linking metabolism and epigenetic regulation emerges as potential GBM therapeutic target.^283^ ERα, a receptor of hormone estrogen and also recognized as a novel RNA-binding protein, controls oncogenic mRNA alternative splicing and translation processes to promote breast tumor cells survival, preventing cells from multiple stressors like ER-stress, immune attack and so on.^284^

RNA-binding proteins are valuable biomarkers for a wide range of gene expression processes, and their regulation of certain oncogenes may have applicability to a class of cancer types driven by these oncogenes. However, RNA-binding proteins are nonenzymatic molecules that lack hydrophobic pockets capable of binding small-molecule inhibitors. Therefore, whether these proteins can become effective targets is yet to be explored.

#### RNA modifications in nervous systems

RNA modification (epitranscriptomics) plays a key role in nervous system development, neuroplasticity and tumorigenesis by dynamically regulating gene expression. For clinical applications, DNA methylation has been used to classify CNS tumors.^285^ RNA modification plays an important role in the interaction between nerves and tumors (rightly falling into the concept of cancer neuroscience), including the regulation of tumor-related neural invasion, neurotrophic signal transduction, and tumor microenvironment remodeling.

m^6^A is abundant and modulates various neural functions. m^6^A promotes translation of target transcripts by YTHDF1 in a neuronal-stimulus-dependent manner, and facilitates learning, memory, and synaptic transmission in the adult mouse hippocampus.^286^ There is a direct interplay between YTHDF1 and Fmr1 (the fly homolog of FMRP) to inhibit the translation of axonal growth transcripts, thus suppressing axonal growth.^287^ m^6^A eraser FTO is enriched in axons, which decreases m^6^A modification of GAP-43 mRNA and promotes its translation, thus giving rise to axon elongation to achieve neuronal development.^288^ m^6^A modification of lncRNA Dubr regulates the axonal elongation and migration of dorsal root ganglion neurons by stabilizing the YTHDF1/3 complex.^269^ The antagonistic function between YTHDF1 and FTO has been noticed during axon growth and development. It is worthy to explore the function between YTHDF1 and FTO in neural remodeling in the tumor tissue. Possibly, the growth of axons is perhaps controlled distinctly in different damage conditions or in different recovery mechanisms, or it may be due to tissue-specific existence, and all of these require further investigation to lead to some therapeutic clues. Based on a variety of database analysis, m^6^A is highly associated with the malignant development of head tumors.^289^ It is possible that tumor cells mimic this RNA modification mechanism to hijack the mechanisms of neural development and form neural infiltration and neural-tumor synapses, which promote tumor cells survival and invasion.

Higher m^1^A modification level has been observed in the major ganglia of simple defensive reflex trained animals than naïve animals, and correlated with increased polyglutamine synthesis and excitability in neurons.^290^ It indicates that RNA modifications may have the capability to establish synapses circuit to learning.

Ψ modified neurotrophin-3 (NT-3) mRNA is delivered into primary Schwann cells to enhance dorsal root ganglia neurite extension,^291^ indicating its potential capability of improving peripheral nerve regeneration. In the neuronal nuclei of the Drosophila brain, numerous RNA editing sites exist, each exhibiting distinct characteristics across different neuronal populations.^292^ These sites vary in editing levels for specific transcripts and often present as unique combinations of multiple editing sites. There may be a complex targeted regulation of editing levels in key transcripts, such as regulating the differentially expressed RNA-binding proteins and other regulators as well as molecular mechanisms.

To date, there is still limited information available on the role of RNA modifications in the progression of the nervous system-tumor axis, and the underlying signaling pathways are currently poorly understood.

#### RNA therapeutics

In view of the unparalleled characteristics of RNA, manipulating RNA biology can show great advantages within the scope of accessibility. RNA can induce protein expression process more rapidly than gene sequence. Meanwhile, RNA-based therapeutics can achieve a local delivery strategy to avoid the invasive body caused by systemic administration.

The structural diversity of RNA enables it to perform diverse biological functions. RNA editing, alternative splicing and RNA modifications expand their functional repertoire. Actually, the mRNA-based genome editing has also been successfully applied to stem cells for many diseases treatments.^293^ ZFN mRNA reserves multilineage potential and low cytotoxicity compared to TALEN mRNA and CRISPR/Cas9 editing, and such editing approach is used in a large animal model to modulate repopulating cells.^294–296^ The mRNA-based iPSC technology delivers Cas9 in the form of mRNA on the basis of the principle of CRISPR/Cas9 system to promote the production of protein molecules, and iPSCs can further differentiate into specific cell types for clinical application.^297–300^ For the current clinical treatment, whether iPSC-induced differentiation can help repair the diseased cells or damaged neurons present in cancer patients, thereby accelerating the recovery of cancer patients.

The limitations of RNA, including instability and off-target efficacy, are current major challenges in clinical application.^301^ We set out to summarize the central role of implementing mRNA-based therapeutics in potentially promising cancer therapy based on the following basic aspects:

(I) RNA is a promising diagnostic marker and therapeutic target: RNA molecules, whose expression levels change during tumor-nervous system interactions, can be used as potential biomarkers for early detection and prognostic assessment of cancer. Single-cell RNA sequencing technology can be used to analyze RNA profiles in tumor samples to provide specific medical treatment. Next, the tumor-nervous system interaction is blocked by silencing neurotrophic factors (such as BDNF) or neurotransmitter synthases (such as IDO1) by non-coding RNA.

(II) RNA can be a versatile therapeutic or preventive drug: mRNA vaccines, such as those against COVID-19, have demonstrated superior effectiveness in vaccine development. Encodes tumor-associated neuro-antigens (such as GDNF) that activate specific T-cells to clear the neuroinvasive microenvironment.

(III) mRNA-based therapies may have less genetic risk and are easy to deliver effectively: Generally, mRNA-based therapies do not introduce gene insertions, deletions, or mutations, thereby reducing genetic risk. Efficient transfection of mRNA transcripts is often easier to achieve than DNA, and entry into the nucleus is not a necessary step for most mRNA transcripts.

##### RNA vaccines and drugs

With the rise of cancer neuroscience, it is worth focusing on how RNA therapies can interfere with the interaction between tumors and the nervous system, including inhibiting tumor-associated neural infiltration, blocking neurotrophic signaling, and inhibiting perineural invasion.

**Fig. 8:**
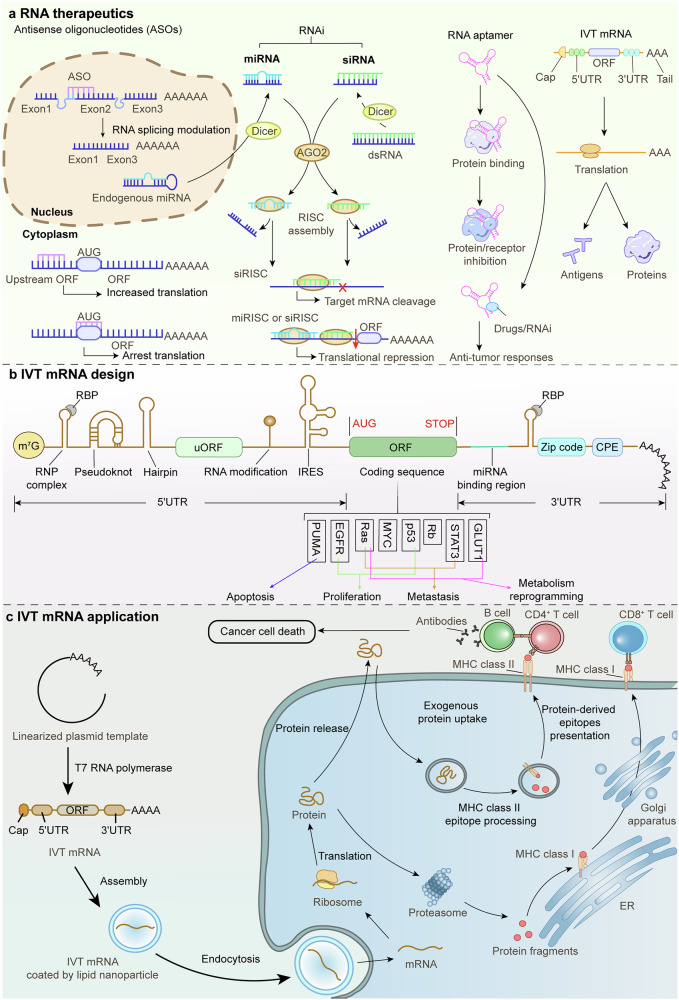
RNA-based cancer therapeutics and the application of in vitro transcribed (IVT) mRNA in antitumor treatment. **a** RNA therapy includes oligonucleotide therapeutics, such as ASOs, siRNAs, miRNAs, and RNA aptamers, which interact with RNA or proteins to regulate gene expression, and IVT mRNAs that translate into functional proteins to serve as an RNA drug/vaccine. **b** The components of IVT mRNA contain 7-methylguanosine (m^7^G) 5′ cap structure, 5′ untranslated region (UTR), open reading frame (ORF), 3′ UTR, and 3′ poly(A) tail. There are RNA structures in UTR, such as pseudoknots and hairpins; sequence binding sites including RNA-binding proteins (RBPs), RNA modification, and non-coding RNA-binding sites. Zip code regulates RNA localization, and CPE regulates RNA stability. ORF codes sequences to control cancer cell fate. **c** IVT mRNA is coated by delivery carriers and taken up by antigen-presenting cells, participating in CD8^+^ T-cell activation by MHC class I, and CD4^+^ T-cell activation by MHC class II. B cells activated by CD4^+^ T-cells produce antibodies, together with cytotoxic CD8^+^ T-cells to kill cancer cells (The arrows represent induce, and the horizontal lines represent inhibit)

Cancer vaccines acting as personalized cancer treatment almost deal with unique composition of mutations in each tumor, and the technology is continuously showcasing great progress towards maturity for clinical application.^302–304^ The development of mRNA-based therapeutics requires the following steps: mRNA design, synthesis, entrapment, pharmacodynamics, pharmacokinetics, safety evaluation, manufacturing, and clinical trials (Fig. 8).^305–307^ Given the characteristics of the cancer-nervous system interaction, it is worthy of discussion that RNA vaccines can intervene in this interaction. We hypothesize the following mechanisms that need to be demonstrated by research: (I) targeting tumor-associated neuro-antigens with vaccines to induce an immune response to clear tumor cells, thereby reducing the negative effects of tumors on the nervous system, (II) the vaccine directly targets nervous system signals in the tumor microenvironment, such as neurotransmitter receptors or related signaling pathways, (III) the above vaccines perhaps bear side effects on the normal nervous system, such as autoimmune reactions and neuroinflammation.

The protein encoded by mRNA vaccine is regarded as an antigen after injecting into antigen-presenting cells, then activates antigen-specific cytotoxic immunity by CD4^+^ T-cells/CD8^+^ T-cells and also humoral immunity by B cells to achieve noticeable treatment.^308,309^ For example, DC-based mRNA vaccines have been shown to prolong GBM patient survival.^310,311^ mRNA vaccines encoding immunostimulants, such as cytokines and chemokines, have shown potential for promoting the maturation and activation of immune cells, thereby improving the tumor microenvironment.^312–314^ In central nervous system tumors, we can target at the level of neural cell differentiation. For example, gangliosides are key proteins in the central system that regulate the growth and protrusion of nerve cells. Among them, GD2, one kind of gangliosides, is highly expressed on the surface of various cancer cells and is a marker of glioblastoma.^315^ Existing clinical studies have shown that anti-GD2 monoclonal antibody drugs can effectively inhibit central nervous system recurrence of neuroblastoma after first complete remission.^316^ In addition, induced pluripotent stem cell (iPS) derived natural killer T (IPS-NKT) cells combined with anti-GD2 monoclonal antibody could effectively inhibit the growth of neuroblastoma.^315^ As potential molecules, mRNA vaccines encoding the GD2 antigen could be explored to determine whether they induce specific T-cell immune responses to eliminate tumor cells. A similar molecule (both responsible in the nervous system and as a tumor marker) is PSMA of prostate cancer cells, and also possesses therapeutic potential worthy of targeting.^317^

If RNA vaccines disrupt the synaptic connections between tumors and neurons by encoding neuron-related antigens specifically expressed on the surface of tumor cells, such as GDNF and NLGN3, it will be a great advance in RNA vaccines. RNA vaccines targeting glutamate metabolism, such as those encoding xCT transporter inhibitors, can block glutamate-cystine exchange resistance to ferroptosis in tumor cells.^318^ It also suggests that RNA vaccines targeting glutamate metabolism may reduce the excitotoxic damage caused by glutamate over-release and subsequent excitatory membrane receptor activation in neurons.^319^ Another valuable application of mRNA-based therapeutics is the delivery of mRNA molecules to regulate neuronal activity.^320^ Neuronal activity is closely linked to the growth and spread of cancer cells, thus modulating this activity could potentially inhibit tumor growth. However, few studies on the delivery of mRNA-encoded neural signaling molecules to treat tumor development have been documented at present. Ultimately, our understanding of the neural circuit-tumor mechanism is limited, and needs future attention and study.

RNA drugs can also include antisense oligonucleotides (ASOs), RNA interference, CRISPR/Cas systems, and RNA aptamers.^321–323^ miR-10b, a unique oncogenic miRNA with high expression in GBM. miR-10b ASO treatment results in targets’ depression, and inhibits GBM growth and progression.^324^ The ability of these RNA drugs to elicit emotional responses in the brain is noteworthy, and the method of successful delivery is also worth attention to promoting RNA therapy. Nanoparticle delivery system is also surprisingly suitable for ribonucleoprotein complexes based on CRISPR technology and aptamer for GBM therapy.^325–327^ Especially, CRISPR/Cas13-mediated RNA editing would dominate in RNA drug-based cancer therapy in future.

Notably, the Nobel Prize in Physiology or Medicine in 2023 was awarded for discovery of RNA modification that enabled the rapid development of successful mRNA vaccines against COVID-19, perhaps indicating the great promise of RNA prevention and therapy strategies for many other diseases including tumor and chronic inflammation. RNA-based therapeutics have also been practiced in CNS diseases. In diffuse midline gliomas (DMGs) that are pediatric high-grade brain tumors, 2’-O-methoxyethyl phosphorothioate ASOs have been demonstrated to inhibit tumor growth, enhance neural stem cell differentiation into neurons, oligodendrocytes, and astrocytes, and promote survival.^328^ TUG1-PRC2 complex inhibits the expression of neuronal differentiation genes, and ASOs targeting TUG1 induce glioma stem cells differentiation and in vivo growth.^329^ These results indicate that neuroscience plays a big part in RNA-based cancer therapy. If the complete production route of mRNA-based drugs can be generated, it is easy to scale up production through chemical synthesis.

In conclusion, future research needs to accurately screen the key molecules of tumor-nerve interaction (such as neurotransmitter receptors and synaptic adhesion proteins) to avoid unwanted damage to normal nerve tissues and interfere with their functions. At the same time, the blood-brain barrier-penetrating LNP or viral vectors should be developed to improve the delivery efficiency of mRNA in the central nervous system. Thus far, only a few phase I clinical trials (e.g., NCT04573140)^330^ have evaluated the safety of RNA vaccines in glioma, which may need further investigation with enlarged sample size and/or combination therapies (e.g., immune checkpoint inhibitors, denervated drugs). Since recent technological innovation has enabled mRNA to become a promising therapeutic tool in the field of cancer therapy, with strong confidence, we expect its application in cancer therapy.

### Cytoskeletal changes

Mutations and abnormal expression of the cytoskeleton and cytoskeletal-associated proteins potentially regulate cancer metastasis and drug resistance. Catecholamines activate FAK signaling pathway via adrenergic receptors to inhibit ovarian cancer cells anoikis, one kind of cell death modes regarded as a type of apoptosis defined in terms of alternations in the cytoskeleton.^331,332^ Similar condition in the nervous system, efficient synaptic transmission is accomplished through a series of interactions with intracellular scaffold proteins, transmembrane helper subunits, adhesion proteins, and other extracellular elements.^333^ Cytoskeletal dynamics also control axon growth during development and axon regeneration.^334^ Next, we discuss the important regulatory roles of cytoskeleton components in cancer cells and in the nervous system, aiming to encourage breakthroughs in this area by extensively studying molecules with direct or indirect linkage to the cytoskeleton.

#### Microfilament

Cancer cells often exhibit aberrant actin cytoskeleton dynamics and altered expression of tropomyosin isoforms, which can contribute to their invasive phenotype. F-actin assists TNT-like protrusion growth.^335^ The actin-supporting TNTs act as open-ended conduits to directly connect cytoplasm of two cells for cargo transportation, including small ions, molecules, as well as large organelles, such as lysosomes and mitochondria.^336^ TNTs have been found in various cancer types including gliomas and non-brain-derived tumor and promote resistance against cytotoxic therapies, just like the function of tumor microtubules network in gliomas.^337^ In a recent report, mixed lineage kinase 3 (MLK3) has been shown to remodel actin cytoskeleton via the MLK3-EPS8 signaling pathway to promote GBM cells migration and invasion.^338^ Actin regulator protein EPS8 has early been proven to inhibit filopodia formation in neuron and promote TNT formation.^339^ Moreover, the upregulation of proteasomal target EPS8 hyperactivates RAC signaling in neurons, leading to actin cytoskeleton alternation and protein kinase JNK activation, which contributes to decreased lifespan.^340^ The protuberant-like morphological changes of these neurons should be an important physical scaffold for the neuro-glioma connection and worthy targets for glioma.

Tiam1, one kind of GEF, orchestrates actin cytoskeleton reorganization and synaptic receptor NMDAR stabilization to control synapse structure and plasticity in anterior cingulate cortex neurons.^341^ Probing whether Tiam1 can modulate neuropathic pain by regulating synaptic connections in the anterior cingulate cortex neurons could provide insights into the mechanisms of pain transmission and potential therapeutic targets. Similar to the role of visual experience in neurofibromas, nociceptive neural pathways may also serve as effective neuro-cancer circuits to suppress cancer. In functional synaptic transmission, receptors must be aggregated before the release site of neurotransmitters, indicating that receptor movement at synapses is a dynamic balance of interactions with synaptic, extra-synaptic, and intracellular receptors.^333^ The secreted BDNF increases synaptic current strength and thus, scientists evaluated whether the increased capture of BDNF by the diffusion of AMPAR receptors enhances the electrical signal.^342^ Assessing receptor movement may find a clue to describe the mechanism of neuro-glioma signal transmission. Ttfields, a non-wound damage device on brain surface, produces alternating electric fields with low intensity and medium frequency.^7^ The electric field force mainly acts on two proteins with high dipole moments (tubulin and septin) in tumor cells to inhibit tumor cell mitosis and DNA damage repair and increase DNA replication pressure. It has already been applied in the clinical treatment of GBM,^7^ demonstrating the important regulatory role of electrical signals in the brain.

However, receptor movement at synapses is a complex process, and the intracellular receptor recycling process extensively related to actin and molecular motors as well as ATP consumption, which constitute complex and discrete signaling pathways. Exploring how these precise molecular mechanisms by which paracrine signaling molecules (such as BDNF and NLGN3) regulate the synaptic transmission and localization of cytoskeletal proteins may define new therapeutic approaches for gliomas.

#### Gap junction

Gap junctions in glioma cells play a pivotal role in the communication between tumor cells and the surrounding neuronal environment, amplify potassium-evoked depolarizing currents in response to neuronal action potential, and promote neuronal activity-evoked calcium transients through neuron-to-glioma network. A subset of gap junction cells receives synaptic input, and the network integrates into brain through synapses and electrical signals.^53^ These unconnected glioma cells can hijack multiple traits of neuronal development via complex calcium signals from neurogliomal synapses by AMPAR, leading to the de novo formation of tumor microtubule network in glioblastoma sub-population, and then promoting cancer cells invasion as well as therapeutic resistance.^53,343^ Synaptic pathophysiology is not limited to primary brain cancer, which also exists in breast-to-brain metastatic tumors. The metastatic tumor to brain establishes glutamatergic synapses mediated by NADMR to integrate in neuronal circuits.^54^ Actin is an important component of cell cytoskeleton to form synaptic and protuberant structures, which is not only involved in the growth, metabolism and migration of normal cells and cancer cells, but also are critical for the connection of nervous system circuits. Altogether, gap junction and actin can be regarded as the key research object of cancer neuroscience.

#### Microtubule

Normally, the two cytoskeletal systems, including microfilaments and microtubules, regulate cytoskeletal structure to control cell motility and other cellular events with a large number of common or even overlapping signaling pathways, such as Rho GTPase pathways.^344^ Likewise, microtubules are also the major components of neuron cytoskeletal, and also essential for many fundamental neuronal and developmental processes, including neuronal migration, polarity, and differentiation, through guiding intracellular transport and inducing morphological changes.^345^ Inhibition of tumor survival by targeting tubulin responsible for axon guidance is validated. Overexpression of tubulin beta 2B class IIb (TUBB2B) in triple-negative breast cancer cells can activate astrocytes, thereby up-regulating the expression of TUBB2B in cancer cells.^346^ Eukaryotic translation elongation factor 1α1 (eEF1A1) is a mediator. TUBB2B eEF1A1 interacts with each other to promote protein synthesis by stabilizing this translation elongation factor. It suggests that there is a positive feedback interaction between TUBB2B and astrocytes in triple-negative breast cancer cells to promote the colonization of brain metastases.^346^ This provides a basis for exploring the mechanism between the expression of axon guidance genes and tumor brain metastasis.

#### Intermediate filament

Intermediate filaments formed by 40 different subunit proteins, including keratins, neurofilaments, desmin, laminin and vimentin, and intermediate filaments link from the ECM to the cytoplasmic interior surrounding the nucleus which allows intermediate filaments to relay information from cell surface to cellular compartments, thus coordinating cytoskeletal activities.^347^ Nestin, an intermediate filament protein, exhibits high expression levels in various cancer types, and is also correlated with aggressive growth, invasion, and poor prognosis.^348,349^ Meanwhile, nestin negatively regulates ACh-induced CDK5-dependent ACh receptor clusters dispersion of the neuromuscular junction synapse, which is essential for postsynaptic differentiation.^350^ In nestin-GFP transgenic mice model, the dentate gyrus of radial glia-like stem cells can ensheathe local synapses and vasculature in the inner molecular layer.^351^ These outcomes indicate that nestin plays a potential role in modulating neuro-tumor synapses formation and tumor migration. Vimentin is another intermediate filament protein that is involved in cancer progression.^352–354^ Loss of vimentin results in peripheral nerve hypermyelination mediated by axonal neuregulin-1 type III.^355^ Acting as a key component of intermediate filaments, vimentin is a fibrous component in both Schwann cells and neuron cytoskeleton, the expression of which is timely and spatially regulated during development and regeneration. Hence, above-discussed researches render sufficient evidence for multiple filament proteins that are involved in cancer neuroscience.

#### Targeting cytoskeletal proteins for therapy

It is now accepted that cytoskeletal components play important roles in the malignant development of cancer cells and the development and differentiation of neurons. Therapeutic strategies developed for brain tumors may have novel prompts for other cancer types. Some therapeutic modes have emerged from features of neuronal function and neurodevelopment, such as preventing neurite-like process growth, blocking neuronal inputs and neurotransmitter signals, and depletion of neuronal subsets in tumor microenvironment. The inhibition of neurite-like protrusions is indeed discovered to reduce tumor cells metastasis.^356,357^ Whether they play a role in the treatment of peripheral tumors remains to be established. This is mainly because abnormalities in neural subsets of extracranial tumors may lead to treatment failure.

Unlike the majority of cells in a single tumor, neuronal subsets of epithelial tumors are very diverse in gene expression and gene regulatory networks,^173,358^ which may require distinct and/or multiple therapeutic approaches. The presence of complex subgroups within the same tumor may require combination therapy targeting a single subgroup. Gap junction inhibitors might be used to disrupt tumor growth and resistance signals, and prevent the downstream effects of synaptic input.^2,3^ In combination with cytotoxic treatment might target effectively to gap junction in brain tumors. Gap junction inhibitors have been found in preclinical models to elevate sensitivity of radio- and chemo-therapy in brain tumor cells.^359^ Meclofenamate and tonabersat are regarded as promising candidate drugs in future clinical trials, which both inhibit the formation of heterocellular astrocyte-carcinoma gap junctions, then preventing brain metastasis and enhancing chemotherapy responses in mouse models.^360^ Synapses also act as the communication structure between neuron-tumor cells. Inhibitors of AMPAR and NMDAR are used to target neuro-glioma synapses in CNS.^2,3^ Perampanel, an AMPAR inhibitor acting as antiepileptic drug, together with radio- and chemo-therapy shows great tolerability and prolonged survival of glioma patients in the phase II clinical trial.^361^ Similarly, recent study finds NMDAR blocker, esmethadone, for the treatment of major depressive disorder with no neurotoxicity in rat models, as well as favorable safety and tolerability in phase 1 and phase 2 clinical trials.^362^ Extending clinical studies of these drugs into therapy of gliomas would be considered a great progress to send the drugs for application of cancer neuroscience knowledge as a core research and development.

## Current challenges in cancer neuroscience

The field of cancer neuroscience is at its infancy, limited successes primarily aggregated on relatively easy parts, such as how the nervous system contributes to the tumorigenesis, growth, spread, recurrence and treatment resistance. Many more areas and related molecular mechanisms, such as how cancers respond to neuronal signals and how peripheral neurons may be widely related to tumors, require research attention.

Cancer heterogeneity is often manifested in gene, epigenetic, transcriptional, protein, and metabolic levels. Even if our current studies can improve the specific targeting of drugs and reduce side effects,^363^ there is still a lack of suitable heterogeneous biomarkers. Current research on tumor neurobiology chiefly relies on static cell models and mouse tumor transplantation experiments, which is difficult to dynamically capture the spatial-temporal evolution of neuro-tumor interactions, and species differences (such as the differences between mice blood-brain barrier and glial interactions with humans) limit the clinical transformation potential. In addition, most studies focus on a single signaling pathway and lack multiomics integration (such as joint analysis of spatial-temporal transcriptome and metabolome), which cannot resolve the spatial heterogeneity of nerve signals in the tumor microenvironment. Spatial multiomics approaches integrate spatial lipidomics and spatial transcriptomics sequencing technologies, achieving the accurate analysis in the areas of metabolism, lipid group and transcriptome data in a tumor tissue sample; associating the network of metabolites/lipids and upstream regulatory genes; characterizing the metabolic regulation and interaction of tumor cells, immune cells and stromal cells in the local tumor microenvironment in situ.^364^ The most important aim is that the combination of scRNA-seq and spatial multi-omics can analyze the distribution of neurotransmitter receptor subtypes. They provide innovative and effective methods, tools and new perspectives for the in-depth study of tumor metabolic heterogeneity.

Despite recent rapid and marvelous advancements, current technological techniques (e.g., neuroimaging) may not fully capture the dynamic changes occurring in the nervous system during cancer development and progression. Techniques to directly record and manipulate brain activity, such as deep brain stimulation (DBS) and optogenetics, have shown potential for treatment of certain diseases, but their applications are limited and carry certain risks. The high-resolution and direct imaging of neuronal activity has been proved as a noninvasive neuroimaging method, which enables direct imaging of neuronal activity with millisecond precision.^365^ ChR2/Halo, an optogenetic tool, enables accurate analysis and localization of neural activity within tumors.^366^ Changes in the expression of photosensitive proteins can visualize changes in neuronal activity and interaction. Moreover, calcium signal and axon dynamics were tracked in real time by living two-photon imaging.^367^ The breakthrough of technical limitations will benefit our research on monitoring the temporospatial dynamics of neural networks in cancer neuroscience.

Access to comprehensive clinical data, particularly concerning the neurobiological aspects of cancer progression and treatment response, remains restricted. Lacking comprehensive clinical data regarding the neurobiological aspects of cancer progression and treatment response hampers the ability to draw robust conclusions and develop effective interventions.

Existing models may not fully encapsulate the complexities of the tumor microenvironment and its influence on neural activity. Mice and nonhuman primate models have played an important role in studying the nervous system, but they differ physiologically and anatomically from humans, which may somewhat complicate the transferability of findings. Transplanting the human dorsal root ganglion into immunodeficient mice to establish a humanized neuro-tumor model may be more closely reflect the human-specific neuro-invasion mechanism, although its survival time will probably be limited by rejection. In order to overcome these limitations, a three-dimensional organoid co-culture system (integrating neurons, glial cells or immune cells and tumor cells) can be constructed in the future to simulate the interaction between synaptic formation and metabolism in vivo. 3D organoid co-culture technology may reduce the dependence on laboratory animals.^368^ The construction of a dorsal root ganglion-tumor organoid could simulate pain nerve signals and tumor growth.^369^ The development of in vitro technology will reduce the pressure of using laboratory animals to some extent, which is worth looking forward to the expansion direction. Given the interactive nature of cancer neuroscience, there is an urgent need to develop experimental models that characterize interactions between tumors, nerves, and other systems.

## Conclusion and perspective

We have witnessed that over the recent decade cancer neuroscience has made steady progress and the hope is to connect the advances to clinical setting to benefit numerous patients with cancer. As such, tools used in neuroscience research and/or cancer therapy are also powerful for understanding cancer pathophysiology: whether the neuroscience-guided cancer therapy meets the target goal. Continued development of advanced neuroimaging techniques, such as functional MRI, diffusion tensor imaging (DTI), and positron emission tomography (PET), to better visualize and understand the dynamic changes in the nervous system associated with cancer progression. Integration of imaging data with other omics data can enhance the understanding of neurobiological mechanisms underlying cancer. In addition, utilizing multi-omics technologies, including genomics, transcriptomics, proteomics, and metabolomics, together with patch clamp technology, to comprehensively characterize the molecular signatures of cancer-neurobiology interactions. This holistic approach can provide insight into underlying mechanisms and identify novel therapeutic targets. Finding suitable biomarkers is critical, which ensure the connection between positive study results and the desired target engagement. Developing imaging and electrophysiological biomarkers may deepen our understanding of the molecular and cellular mechanisms underlying neuron-cancer crosstalk.

Furthermore, targeting the neural pathways and structures in tumor tissue requires careful validation of neurocognitive effects and behavioral assessment. Targeting neural-cancer interactions is highly likely to be used as a sensitizer for radiotherapy or chemotherapy. The mutation status of the genes may affect the characteristics of certain tumors, such as aggressive and recurrent tumors.^18^ Therefore, it is important to devise the optimal combination regimen with the concurrent use of cytotoxic, epigenetic, or immunotherapeutic therapies.

Moreover, artificial intelligence has already made progress in accelerating drug discovery and successfully using genomic and chemical data to predict drug behavior rather than using large-scale screening assays may accelerate drug reutilization.^370^ It is feasible and wise to take advantage of the rise of new technologies and apply them to research and practice. Leveraging bioinformatics tools and computational modeling approaches to analyze large-scale omics data, identify biomarkers, and predict patient outcomes. Integrating computational models of cancer-neurobiology interactions can facilitate hypothesis generation and accelerate drug discovery efforts. Exploring the adaptive response of the nervous system to cancer-related stressors, includes neuroplasticity mechanisms and resilience factors. Identifying neural pathways associated with resilience can help in the development of interventions to improve mental health and quality of life of patients during cancer treatment.

One may consider how peripheral drug delivery can enhance targeting, and how intracranial tumor drug delivery can cross the blood-brain barrier and decrease toxic side effects, which has been perplexing humans for a long time.^371,372^ When using drugs guided by neuroscience to act on tumors, screening the appropriate dose of drugs, such as reducing short-term side effects (vomiting, mood disorders, and suicidal tendencies) and long-term physical damage (hormone disorders, and organ failure), are the key factors to be aware of whether they can be used in the clinical practice. Under the philosophy of cancer neuroscience, it is expected that the incorporation of RNA-based therapeutics would provide new treatments for individuals suffering from cancer and improve the efficiency of existing treatments.
